# Epidemiological analysis of turner syndrome in children aged 0–14 years: global, regional, and national perspectives (1990-2021)

**DOI:** 10.3389/fendo.2025.1552300

**Published:** 2025-04-30

**Authors:** Fang Ding, Jiaoli Xu, Jingxuan Xiong, Qinhong Li, Zugen Cheng, Lili Deng

**Affiliations:** ^1^ Department of Cardiology, Kunming Children's Hospital, Kunming, China; ^2^ Department of Respiratory Medicine, Kunming Children’s Hospital, Kunming, China; ^3^ Department of Cardiology, The First Affiliated Hospital of Kunming Medical University, Kunming, China

**Keywords:** turner syndrome, GBD (global burden disease), prevalence, DALYs - disability-adjusted life years, children

## Abstract

**Background:**

Turner syndrome (TS), a chromosomal disorder (45, X) affecting approximately 1 in 2,000 female births, results in multisystem morbidity. This study aims to report global trends in the prevalence and disability-adjusted life years (DALYs) of childhood TS from 1990 to 2021.

**Methods:**

This study analyzed the prevalence and DALYs of TS in children aged 0–14 years using data from the Global Burden of Disease (GBD) database. Prevalence and DALYs were calculated per 100,000 population with 95% uncertainty intervals (UI). A log-transformed linear regression model was applied to estimate the average annual percentage change (EAPC) and evaluate temporal trends.

**Findings:**

Globally, the prevalence of TS in children in 2021 was 240598.45 cases (95% UI, 185491.24,318611.47), with 174,186.30 DALYs (95% UI, 127,104.64–223,265.92). From 1990 to 2021, the prevalence increased by 12.81% (95% UI, 11.37% to 14.05%), and DALYs decreased by 66.13% (95% UI, -79.97% to -44.24%). Among the five Sociodemographic Index (SDI) regions, the highest EAPCs were observed in the High SDI regions for prevalence (0.03; 95% CI, 0.01–0.05) and DALYs (0.03; 95% CI, 0.01–0.05). Regionally, the largest decline in prevalence rate occurred in Andean Latin America (EAPC = –0.44; 95% CI, –0.45 to –0.43), and the greatest increase was recorded in the Caribbean (EAPC = 0.05; 95% CI, 0.00–0.10). At the national level, India had the highest number of TS cases in 2021, with 45,941.86 cases (95% UI, 35,104.78–61,429.72).

**Interpretation:**

Overall, these findings provide a critical foundation for shaping public health strategies and policy decisions aimed at reducing the global burden of pediatric TS through improved diagnostic practices, comprehensive care, and targeted interventions.

## Introduction

Turner Syndrome (TS) represents the most frequent sex chromosomal abnormality in girls and women, caused by the missing part or entirety of an X chromosome (1). It is the only viable monosomy syndrome in humans, with an incidence of approximately 1 in 4,000 to 1 in 2,500 live female births (2). TS is primarily caused by the complete or partial loss of one X chromosome, with the most common karyotype being 45, X (3). The clinical manifestations of TS are highly heterogeneous, but the typical phenotype includes short stature, ovarian insufficiency, and congenital cardiovascular malformations (4–6). Studies have reported that about 25% of TS patients are diagnosed prenatally or at birth due to characteristic features such as a webbed neck and other fetal lymphoedema signs (7). Additional primary clinical features of TS include thyroid autoimmune abnormalities (8), congenital cardiovascular anomalies (9), liver abnormalities (10), hypertension (11), hearing loss (12), and renal anomalies (13). The age at diagnosis is predominantly distributed across three periods: under 1 year (14.9%), between 10–17 years (33.2%), and adulthood (38.5%), with a median age of diagnosis at 15.1 years, indicating a significant delay in diagnosis. Despite an increasing prevalence, the incidence rate of TS remains stable (14). Previous research has demonstrated that individuals with TS have a reduced life expectancy compared to the general population, with a difference of up to 12.5 years (15). In summary, TS presents clinicians with a multitude of challenges, including genetic, developmental, endocrine, cardiovascular, psychosocial, and reproductive issues. Patients may experience various complications throughout their lifespans, affecting not only their physical health but also leading to psychological and social impairments, thereby increasing the societal burden.

Currently, there is a paucity of research on the global burden and regional disparities of TS. Understanding the differences in disease burden across various regions can aid in formulating targeted health policies and promoting the rational allocation of medical resources. This study utilizes data from the Global Burden of Disease (GBD) study spanning from 1990 to 2021 to analyze the prevalence and Disability-Adjusted Life Years (DALYs) of children with TS aged 0–14 years at global, regional, and national levels, stratified by location and type. The aim is to assess the impact of socioeconomic factors and healthcare accessibility on the health burden of TS. This will provide references for healthcare professionals to develop new prevention and treatment strategies and offer insights for improving patient care and public health measures.

## Methods

### Overview and methodological details

Directed by the Institute for Health Metrics and Evaluation (IHME) at the University of Washington, the GBD data source aims to quantify health losses caused by various diseases, injuries, and risk factors. It is recognized as one of the most comprehensive and systematic global epidemiological efforts (16). The GBD database was utilized to collect available data on individuals aged 0–14 years diagnosed with TS. Specifically, the dataset encompasses prevalence rates and cases at global, regional, and national levels, as well as DALY rates and cases. Additionally, trends in case numbers from 1990 to 2021 are included. All data employed in this study are accessible for download from the Global Health Data Exchange (GHDx) website (https://vizhub.healthdata.org/gbdresults/). Specifically, in the GHDx query tool, we chose “Cause” as “Turner syndrome”. Due to the lack of racial and ethnic data within the GBD database, analyses of these factors were not performed. This cross-sectional investigation entails examining and characterizing disease data across different timeframes and regions, ensuring that no identifiable personal information is included. As a result, the Ethics Committee of Kunming Children’s Hospital approved a waiver for the informed consent process. The study fully complied with the Strengthening the Reporting of Observational Studies in Epidemiology (STROBE) guidelines (17).

### DALYs

It is a way to quantify the burden of diseases, injuries, and risk factors on a population. The concept combines years of life lost (YLL) due to premature mortality and years lived with disability (YLD) for incident cases of a disease. These metrics are calculated using the following equations:


YLL = Number of deaths × Standard life expectancy at death

YLD = Disease prevalence × Disability weight

Disability weights, determined through expert consensus, scale disease severity on a continuum from 0 (optimal health) to 1 (equivalent to mortality).

### Socio-demographic index

The Socio-Demographic Index (SDI) is a comprehensive measure of socio-economic development for countries and regions, encompassing factors such as economic structure and scale, educational attainment, living standards, and social welfare and protection (18). SDI values range from 0 to 1, with higher values indicating more advanced socio-economic conditions. Utilizing data from the GBD database, countries and regions are categorized into five SDI tiers: low, lower-middle, middle, upper-middle, and high. This stratification facilitates the analysis of how socio-economic status and geographical disparities impact the burden of TS in children.

### Statistical analysis

Prevalence and DALYs rates per 100,000 population, along with their 95% uncertainty intervals, were extracted from the GBD database. A log-transformed linear regression model was employed to estimate the average annual percentage change (EAPC) and its confidence interval (CI), facilitating the analysis of temporal trends in the prevalence and DALYs of TS in children from 1990 to 2021. The EAPC is particularly valuable for assessing long-term trends, as it captures the overall increase or decrease in prevalence rates over time, independent of short-term fluctuations. An EAPC with a lower 95% CI bound exceeding 0 indicates a significant upward trend in the respective metric, whereas an EAPC with an upper 95% CI bound below 0 signifies a significant downward trend. Additionally, fitted curves were utilized to examine the relationship between disease burden indicators and the SDI (19). All analyses were conducted using R version 4.3.3, with statistical significance determined at p < 0.05.

## Result

### Global burden trends

#### Prevalence

**Figure 1:**
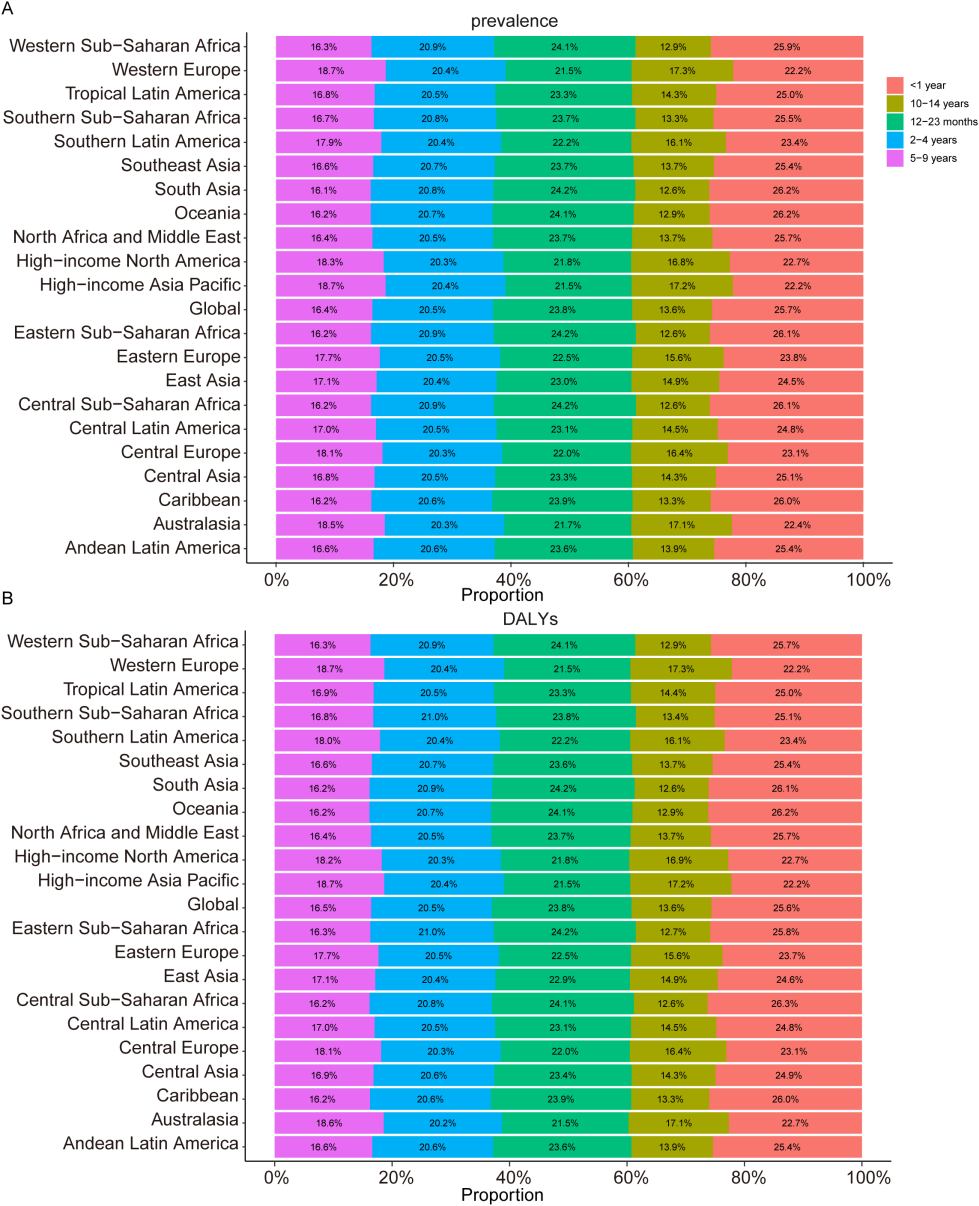
Age-specific Percentages of Childhood Turner Syndrome Prevalence and Disability-Adjusted Life Years (DALYs) in 2021. **(A)** Prevalence. **(B)** DALYs.

In 2021, the global prevalence of TS in children was 240,598.45 (95% UI, 185,491.24–318,611.47), representing a 12.81% (95% UI, 11.37–14.05) increase compared to 1990, when the prevalence was 213,271.59 (95% UI, 164,243.71–282,625.41). The global prevalence rate in 2021 was 11.96 (95% UI, 9.22–15.84), slightly lower than the 1990 rate of 12.26 (95% UI, 9.44–16.25). The estimated annual percentage change (EAPC) was -0.03 (95% CI, -0.06 to -0.00) (**Table 1**). In 2021, the prevalence rates by age groups were as follows: for children under 1 year, 17.75 (95% UI, 13.30–23.84); for children aged 1–2 years, 16.38 (95% UI, 13.32–21.95); for children aged 2–4 years, 14.12 (95% UI, 10.72–19.03); for children aged 5–9 years, 11.31 (95% UI, 8.68–15.18); and for children aged 10–14 years, 9.37 (95% UI, 7.19–12.42) (**Supplementary Figure S1A**). The prevalence rate of TS in children under 1 year of age is the highest, accounting for 25.7% of all children with TS in 2021, whereas children aged 10–14 years accounted for 13.6% of the total TS cases in 2021 (**Figure 1A**).

**Table 1:** Prevalence of turner syndrome in children between 1990 and 2021 at the global and regional level.

Location	1990		2021		1990-2021	
	Prevalence Cases	Prevalence Rate	Prevalence Cases	Prevalence Rate	Cases change	EAPC^a^
Global	213271.59 (164243.71,282625.41)	12.26 (9.44,16.25)	240598.45 (185491.24,318611.47)	11.96 (9.22,15.84)	12.81 (11.37,14.05)	-0.03 (-0.06,-0.00)
High SDI	29527.53 (23665.73,37883.41)	15.89 (12.74,20.39)	27703.64 (22036.55,36140.06)	16.06 (12.77,20.95)	-6.18 (-8.59,-4.12)	0.03 (0.01,0.05)
High-middle SDI	28631.34 (22233.47,38182.65)	10.46 (8.13,13.95)	22300.52 (17417.51,29485.04)	9.66 (7.54,12.77)	-22.11 (-24.25,-19.98)	-0.14 (-0.19,-0.09)
Middle SDI	57166.05 (43725.75,75904.78)	9.90 (7.58,13.15)	53628.09 (41243.20,71192.11)	9.46 (7.28,12.56)	-6.19 (-8.11,-3.87)	-0.10 (-0.13,-0.06)
Low-middle SDI	63383.03 (48126.95,85057.37)	13.43 (10.19,18.02)	71814.40 (55288.99,95011.90)	12.39 (9.54,16.39)	13.30 (9.83,16.60)	-0.23 (-0.24,-0.21)
Low SDI	34397.14 (25975.31,46246.37)	15.03 (11.35,20.20)	64984.99 (49441.90,87103.59)	14.12 (10.74,18.93)	88.93 (84.04,94.99)	-0.20 (-0.21,-0.18)
Regions						
Andean Latin America	1491.06 (1144.03,1954.50)	10.04 (7.70,13.16)	1593.79 (1230.92,2100.19)	8.81 (6.80,11.61)	6.89 (-2.42,18.48)	-0.44 (-0.45,-0.43)
Australasia	503.80 (390.94,656.89)	10.99 (8.52,14.32)	620.40 (485.52,814.88)	10.82 (8.47,14.22)	23.14 (12.42,34.14)	-0.06 (-0.07,-0.04)
Caribbean	1192.43 (910.97,1591.46)	10.45 (7.98,13.94)	1209.24 (928.84,1615.88)	10.51 (8.07,14.04)	1.41 (-4.36,8.62)	0.05 (0.00,0.10)
Central Asia	3654.72 (2842.60,4746.29)	14.62 (11.37,18.99)	4082.86 (3135.89,5373.57)	14.75 (11.33,19.42)	11.71 (5.62,18.58)	0.04 (0.00,0.08)
Central Europe	3119.20 (2440.10,4124.39)	10.58 (8.28,13.99)	1879.34 (1470.82,2457.13)	10.62 (8.31,13.88)	-39.75 (-41.63,-37.49)	-0.01 (-0.06,0.04)
Central Latin America	6209.17 (4799.18,8192.29)	9.64 (7.45,12.72)	5556.31 (4313.19,7363.96)	8.75 (6.79,11.60)	-10.51 (-13.45,-7.48)	-0.27 (-0.30,-0.24)
Central Sub-Saharan Africa	3970.11 (2968.77,5411.70)	15.69 (11.73,21.39)	8545.15 (6370.60,11471.94)	14.56 (10.86,19.55)	115.24 (93.76,139.84)	-0.23 (-0.27,-0.20)
East Asia	25093.62 (19073.52,33754.90)	7.61 (5.78,10.23)	19250.41 (14826.40,25551.69)	7.20 (5.55,9.56)	-23.29 (-26.23,-20.06)	-0.15 (-0.22,-0.09)
Eastern Europe	8399.05 (6551.88,11025.87)	16.32 (12.73,21.43)	5511.32 (4299.56,7364.83)	15.55 (12.13,20.78)	-34.38 (-36.71,-32.11)	0.00 (-0.06,0.07)
Eastern Sub-Saharan Africa	14546.48 (10903.57,19520.84)	16.06 (12.04,21.55)	26977.84 (20446.13,36162.50)	15.12 (11.46,20.27)	85.46 (79.17,93.45)	-0.23 (-0.25,-0.20)
High-income Asia Pacific	4656.00 (3694.90,5987.38)	13.23 (10.50,17.01)	2806.89 (2239.40,3612.22)	12.52 (9.99,16.11)	-39.71 (-43.05,-36.64)	-0.17 (-0.19,-0.16)
High-income North America	13363.67 (10549.36,17420.24)	21.67 (17.10,28.24)	14048.26 (11108.08,18439.41)	21.41 (16.93,28.10)	5.12 (1.68,8.75)	-0.06 (-0.08,-0.03)
North Africa and Middle East	9673.06 (7369.33,12761.93)	6.89 (5.25,9.08)	11884.84 (9231.38,15671.60)	6.48 (5.04,8.55)	22.87 (18.33,28.13)	-0.13 (-0.15,-0.11)
Oceania	320.67 (244.10,424.88)	11.97 (9.11,15.85)	608.10 (452.36,799.67)	11.97 (8.90,15.74)	89.63 (69.03,115.00)	0.02 (0.01,0.04)
South Asia	59935.19 (45412.68,80543.47)	13.83 (10.48,18.59)	64044.92 (49100.92,85301.39)	12.63 (9.68,16.82)	6.86 (2.97,10.40)	-0.25 (-0.27,-0.22)
Southeast Asia	21151.53 (16124.60,27858.15)	12.39 (9.44,16.32)	19415.76 (14929.15,25682.41)	11.25 (8.65,14.88)	-8.21 (-10.66,-5.32)	-0.34 (-0.36,-0.32)
Southern Latin America	2413.77 (1857.64,3226.17)	16.17 (12.45,21.61)	2268.26 (1762.03,3004.96)	15.65 (12.16,20.73)	-6.03 (-14.34,3.69)	-0.07 (-0.09,-0.05)
Southern Sub-Saharan Africa	3354.02 (2501.49,4498.84)	16.21 (12.09,21.74)	3741.99 (2805.00,4965.57)	15.55 (11.66,20.63)	11.57 (6.34,17.80)	-0.11 (-0.14,-0.07)
Tropical Latin America	6487.65 (4980.07,8663.62)	12.10 (9.29,16.16)	6041.57 (4667.70,8081.41)	12.04 (9.30,16.10)	-6.88 (-9.92,-3.42)	0.01 (-0.00,0.02)
Western Europe	10507.81 (8543.34,13081.45)	14.80 (12.03,18.42)	10071.19 (7973.29,12951.92)	14.78 (11.71,19.01)	-4.16 (-8.99,0.29)	-0.05 (-0.09,-0.01)
Western Sub-Saharan Africa	13228.58 (10020.22,17756.16)	15.05 (11.40,20.21)	30440.01 (23122.60,40424.87)	14.17 (10.77,18.82)	130.11 (124.73,135.06)	-0.15 (-0.17,-0.14)

EAPC, estimated annual percentage change; SDI, Sociodemographic Index; UI, uncertainty interval. EAPC^a^ is expressed as 95% CIs.

#### DALYs

In 2021, the global DALYs for TS in children was 3,328.35 (95% UI, 1,344.90–6,251.47), reflecting a decrease of 66.13 (95% UI, -79.97 to -44.24) compared to 1990, when the DALYs amounted to 2,948.47 (95% UI, 1,198.17–5,551.18). The DALY rate in 2021 was 0.17 (95% UI, 0.07–0.31), almost identical to the rate of 0.17 (95% UI, 0.07–0.32) in 1990. The EAPC for DALYs was -0.02 (95% CI, -0.05 to 0.00) (**Table 1**). The DALY rates by age group in 2021 were as follows: for children under 1 year, 0.24 (95% UI, 0.10–0.46); for children aged 1–2 years, 0.23 (95% UI, 0.09–0.43); for children aged 2–4 years, 0.20 (95% UI, 0.08–0.40); for children aged 5–9 years, 0.16 (95% UI, 0.06–0.29); and for children aged 10–14 years, 0.13 (95% UI, 0.05–0.25) (**Supplementary Figure S1B**). In 2021, children under 1 year of age had the highest DALY rate from TS, accounting for 25.6% of total TS-related DALYs among children, whereas those aged 10–14 contributed 13.6% (**Figure 1B**).

### TS in children: regional trends by SDI

#### Prevalence

**Figure 2:**
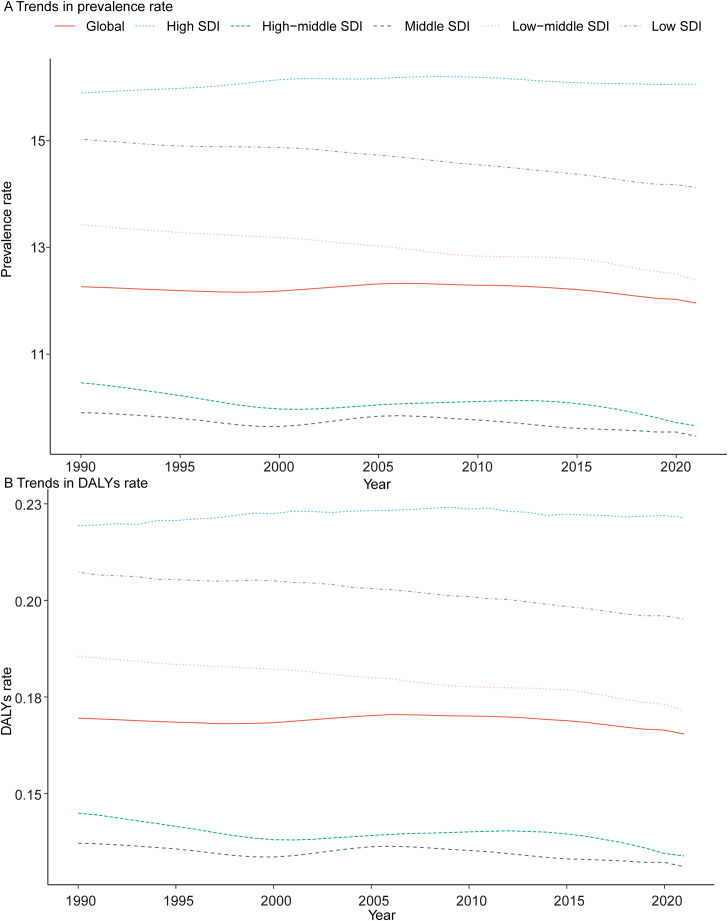
Epidemiologic Trends of Prevalence, and Disability-Adjusted Life-Years (DALYs) Rates in 5 Sociodemographic Index (SDI) Regions of Turner Syndrome in Children From 1990 to 2021. **(A)** Trends in prevalence rate. **(B)** Trends in DALYs rate.

In 1990, the Low-middle SDI region had the highest number of pediatric TS cases, with 63,383.03 (95% UI, 48,126.95–85,057.37), while the High-middle SDI region had the fewest, with 28,631.34 (95% UI, 22,233.47–38,182.65). By 2021, the Low-middle SDI region still had the highest number of cases at 71,814.40 (95% UI, 55,288.99–95,011.90), and the High-middle SDI region again recorded the fewest at 22,300.52 (95% UI, 17,417.51–29,485.04). Additionally, in 2021, the High SDI region had the highest prevalence rate of pediatric TS (16.06 per 100,000; 95% UI, 12.77–20.95), whereas the Middle SDI region had the lowest (9.46 per 100,000; 95% UI, 7.28–12.56) (**Table 1**, **Figure 2A**).

#### DALYs

In 2021, the Low-middle SDI region exhibited the highest number of DALYs for pediatric TS at 993.76 (95% UI, 403.70–1,897.16), while the High-middle SDI region had the fewest at 309.05 (95% UI, 126.28–573.39). The High SDI region recorded the highest DALY rate (0.22 per 100,000; 95% UI, 0.09–0.40), while the High-middle and Middle SDI regions had the lowest rates (both at 0.13 per 100,000). Between 1990 and 2021, among all five SDI regions, only the Low SDI region showed a rise in DALYs for pediatric TS, increasing from 474.50 (95% UI, 192.10–900.91) in 1990 to 898.27 (95% UI, 358.27–1,710.59) in 2021. Notably, only the High SDI region exhibited a positive EAPC (0.03; 95% CI: 0.01–0.05) (**Table 2**, **Figure 2B**).

**Table 2:** DALYs of turner syndrome in children between 1990 and 2021 at the global and regional level.

Location	1990		2021		1990-2021	
	DALYs Cases	DALYs Rate	DALYs Cases	DALYs Rate	Cases change	EAPC^a^
Global	2948.47 (1198.17,5551.18)	0.17 (0.07,0.32)	3328.35 (1344.90,6251.47)	0.17 (0.07,0.31)	-66.13 (-79.97,-44.24)	-0.02 (-0.05,0.00)
High SDI	407.53 (164.77,741.29)	0.22 (0.09,0.40)	382.07 (161.31,683.97)	0.22 (0.09,0.40)	-67.26 (-73.28,-60.72)	0.03 (0.01,0.05)
High-middle SDI	396.57 (160.72,741.40)	0.14 (0.06,0.27)	309.05 (126.28,573.39)	0.13 (0.05,0.25)	-86.04 (-92.20,-76.86)	-0.14 (-0.19,-0.09)
Middle SDI	791.55 (325.06,1483.93)	0.14 (0.06,0.26)	742.90 (302.60,1389.00)	0.13 (0.05,0.25)	-80.51 (-88.63,-68.29)	-0.09 (-0.13,-0.06)
Low-middle SDI	876.01 (355.14,1654.51)	0.19 (0.08,0.35)	993.76 (403.70,1897.16)	0.17 (0.07,0.33)	-48.05 (-71.24,4.10)	-0.22 (-0.24,-0.21)
Low SDI	474.50 (192.10,900.91)	0.21 (0.08,0.39)	898.27 (358.27,1710.59)	0.20 (0.08,0.37)	1.06 (-49.89,144.49)	-0.19 (-0.20,-0.17)
Regions						
Andean Latin America	20.67 (8.52,39.60)	0.14 (0.06,0.27)	22.10 (9.00,41.35)	0.12 (0.05,0.23)	-52.91 (-79.24,-7.38)	-0.44 (-0.46,-0.43)
Australasia	6.96 (2.75,12.81)	0.15 (0.06,0.28)	8.51 (3.58,16.21)	0.15 (0.06,0.28)	-58.44 (-68.65,-45.98)	-0.06 (-0.08,-0.05)
Caribbean	16.51 (6.75,30.96)	0.14 (0.06,0.27)	16.76 (6.73,32.34)	0.15 (0.06,0.28)	-40.23 (-62.62,-10.81)	0.05 (0.01,0.10)
Central Asia	50.59 (20.53,94.78)	0.20 (0.08,0.38)	56.52 (23.37,107.41)	0.20 (0.08,0.39)	-54.66 (-69.54,-33.74)	0.04 (0.00,0.09)
Central Europe	43.21 (17.44,80.08)	0.15 (0.06,0.27)	26.03 (10.67,47.88)	0.15 (0.06,0.27)	-85.17 (-89.00,-79.98)	-0.01 (-0.06,0.04)
Central Latin America	86.03 (34.80,161.42)	0.13 (0.05,0.25)	76.98 (31.35,144.72)	0.12 (0.05,0.23)	-65.64 (-75.07,-51.33)	-0.27 (-0.30,-0.24)
Central Sub-Saharan Africa	54.90 (22.14,104.22)	0.22 (0.09,0.41)	118.39 (45.15,228.60)	0.20 (0.08,0.39)	-8.40 (-47.90,182.93)	-0.21 (-0.24,-0.18)
East Asia	347.42 (143.31,655.93)	0.11 (0.04,0.20)	266.79 (108.65,502.41)	0.10 (0.04,0.19)	-88.96 (-94.81,-80.01)	-0.15 (-0.21,-0.09)
Eastern Europe	116.33 (47.42,217.35)	0.23 (0.09,0.42)	76.38 (31.03,142.47)	0.22 (0.09,0.40)	-85.10 (-87.59,-82.30)	0.01 (-0.05,0.07)
Eastern Sub-Saharan Africa	200.16 (81.55,377.44)	0.22 (0.09,0.42)	372.60 (150.71,711.19)	0.21 (0.08,0.40)	-9.59 (-59.72,132.82)	-0.21 (-0.24,-0.19)
High-income Asia Pacific	64.50 (26.38,117.99)	0.18 (0.07,0.34)	38.88 (16.15,70.55)	0.17 (0.07,0.31)	-78.28 (-82.84,-72.35)	-0.17 (-0.19,-0.16)
High-income North America	183.69 (72.45,339.79)	0.30 (0.12,0.55)	193.03 (79.65,350.02)	0.29 (0.12,0.53)	-60.10 (-64.72,-54.88)	-0.05 (-0.07,-0.02)
North Africa and Middle East	134.01 (54.69,256.29)	0.10 (0.04,0.18)	164.60 (66.48,310.62)	0.09 (0.04,0.17)	-55.12 (-74.47,-13.80)	-0.13 (-0.15,-0.11)
Oceania	4.44 (1.83,8.41)	0.17 (0.07,0.31)	8.41 (3.31,16.05)	0.17 (0.07,0.32)	70.55 (14.07,170.68)	0.02 (0.00,0.04)
South Asia	828.66 (333.04,1582.74)	0.19 (0.08,0.37)	886.44 (360.99,1699.44)	0.17 (0.07,0.34)	-55.78 (-76.34,-4.79)	-0.24 (-0.26,-0.22)
Southeast Asia	292.80 (119.70,550.85)	0.17 (0.07,0.32)	268.93 (107.83,507.94)	0.16 (0.06,0.29)	-49.02 (-70.99,14.13)	-0.33 (-0.36,-0.31)
Southern Latin America	33.47 (13.37,63.41)	0.22 (0.09,0.42)	31.42 (12.57,59.23)	0.22 (0.09,0.41)	-66.14 (-74.74,-54.77)	-0.07 (-0.09,-0.04)
Southern Sub-Saharan Africa	46.34 (18.92,86.88)	0.22 (0.09,0.42)	51.69 (21.20,98.87)	0.21 (0.09,0.41)	-9.08 (-44.06,69.06)	-0.11 (-0.14,-0.08)
Tropical Latin America	89.70 (37.13,168.68)	0.17 (0.07,0.31)	83.56 (34.99,156.64)	0.17 (0.07,0.31)	-61.97 (-72.48,-48.18)	0.01 (0.00,0.02)
Western Europe	145.49 (59.85,267.37)	0.20 (0.08,0.38)	139.46 (57.93,252.99)	0.20 (0.09,0.37)	-67.07 (-71.54,-61.51)	-0.05 (-0.09,-0.01)
Western Sub-Saharan Africa	182.60 (74.09,343.54)	0.21 (0.08,0.39)	420.88 (171.31,798.41)	0.20 (0.08,0.37)	58.32 (-18.90,209.98)	-0.15 (-0.16,-0.13)

EAPC, estimated annual percentage change; SDI, Sociodemographic Index; UI, uncertainty interval. EAPC^a^ is expressed as 95% CIs.

### TS in children: geographic regional trends

#### Prevalence

**Figure 3:**
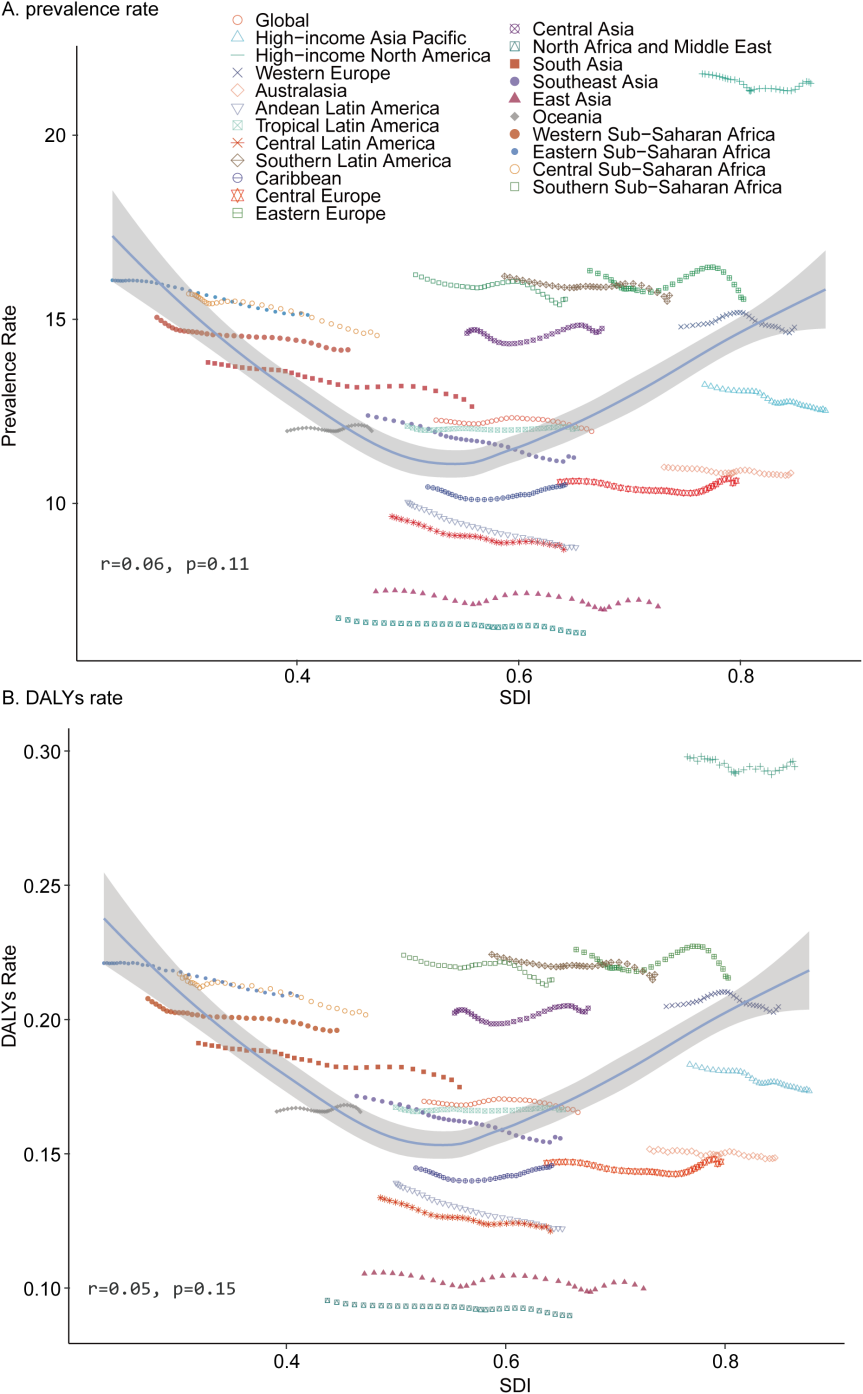
Prevalence and Disability-Adjusted Life-Years (DALYs) Rates for Childhood Turner Syndrome From 1990 to 2021. **(A)** Prevalence rate. **(B)** DALYs rate.

In 1990, Oceania reported the lowest number of pediatric TS cases (320.67; 95% UI, 244.10–424.88), whereas South Asia recorded the highest (59,935.19; 95% UI, 45,412.68–80,543.47). By 2021, Oceania remained the region with the fewest cases (608.1; 95% UI, 452.36–799.67), and South Asia continued to show the greatest burden (64,044.92; 95% UI, 49,100.92–85,301.39). In 2021, the lowest TS prevalence rate was observed in North Africa and the Middle East (6.48; 95% UI, 5.04–8.55), while High-income North America exhibited the highest rate (21.41; 95% UI, 16.93–28.10). Between 1990 and 2021, the largest decline in prevalence rate occurred in Andean Latin America (EAPC = –0.44; 95% CI, –0.45 to –0.43), and the greatest increase was recorded in the Caribbean (EAPC = 0.05; 95% CI, 0.00–0.10) (**Table 1**). In 2021, the global SDI was 0.67. Eight regions had pediatric TS prevalence rates below the global average, whereas thirteen exceeded it (**Figure 3A**).

#### DALYs

In 2021, Oceania recorded the fewest DALYs for pediatric TS (8.41; 95% UI, 3.31–16.05), while South Asia experienced the highest DALYs (886.44; 95% UI, 360.99–1,699.44). The lowest DALYs rate was found in North Africa and the Middle East (0.09; 95% UI, 0.04–0.17), and the highest in High-income North America (0.29; 95% UI, 0.12–0.53). From 1990 to 2021, Andean Latin America showed the greatest reduction in DALYs rate (EAPC = –0.44; 95% CI, –0.46 to –0.43), whereas the Caribbean exhibited the largest increase (EAPC = 0.05; 95% CI, 0.01–0.10) (**Table 2**). Eight regions with a global SDI of 0.67 in 2021 had DALY rates below the global average, and thirteen exceeded it (**Figure 3B**).

### TS in children: national trends

#### Prevalence

**Figure 4:**
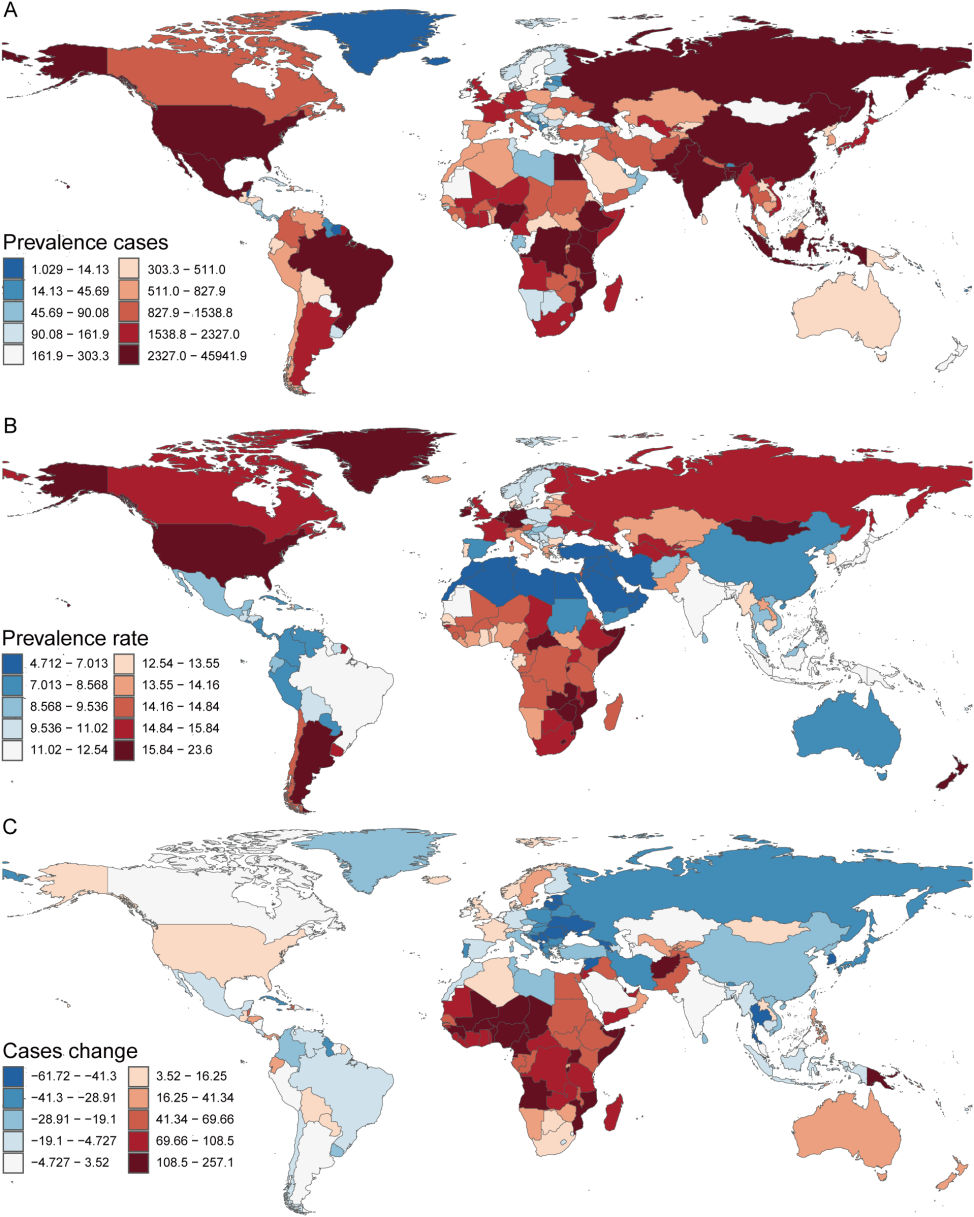
Prevalence of Turner Syndrome in Children in 204 Countries and Territories. **(A)** Prevalence cases. **(B)** Prevalence rate. **(C)** Change in Prevalence cases.

In 2021, among 204 countries surveyed, Niue recorded the lowest number of children with TS (0.04 [95% UI, 0.03–0.05]), whereas India reported the highest (45,941.86 [95% UI, 35,104.78–61,429.72]) (**Supplementary Table S2**, **Figure 4A**). Kuwait exhibited the lowest prevalence of pediatric TS (4.71 [95% UI, 3.59–6.20]), while New Zealand had the highest (23.60 [95% UI, 18.38–31.00]) (**Supplementary Table S2**, **Figure 4B**). Between 1990 and 2021, the largest decline in pediatric TS cases was observed in the United States Virgin Islands (case change = –61.72 [95% UI, –67.17 to –56.18]), whereas Qatar displayed the greatest increase (case change = 257.12 [95% UI, 214.03–315.98]) (**Supplementary Table S2**, **Figure 4C**). In terms of prevalence trends during this period, Equatorial Guinea experienced the steepest decrease (EAPC = –0.96 [95% CI, –1.04 to –0.89]), whereas France showed the largest rise (EAPC = 0.20 [95% CI, –0.05 to 0.45]) (**Supplementary Table S2**). Globally, the pediatric TS prevalence in 2021 reached 11.96 (95% UI, 9.22–15.84) per 100,000 individuals; among the 204 countries assessed, 99 fell below this global benchmark, while the remainder exceeded it.

#### DALYs

In 2021, India recorded the highest number of DALYs attributable to pediatric TS, at 635.79 (95% UI, 255.73–1220.19), whereas New Zealand exhibited the highest DALY rate, reaching 0.32 (95% UI, 0.12–0.63). Over the period 1990–2021, the largest decrease in DALYs was observed in the United States Virgin Islands (case change = –61.69 [95% UI, –67.17 to –56.18]), while Qatar showed the greatest increase (case change = 257.07 [95% UI, 214.03–315.98]) (**Supplementary Table S1**, **Supplementary Figures S2A–C**). Concerning the DALY rate, Equatorial Guinea experienced the most pronounced decline (EAPC = –0.93 [95% CI, –1.00 to –0.86]), whereas France had the largest rise (EAPC = 0.20 [95% CI, –0.06 to 0.45]) (**Supplementary Table S1**). Globally, the DALY rate for pediatric TS in 2021 was 0.17 (95% UI, 0.07–0.31). Among the 204 countries analyzed, 98 had DALY rates below this global average, 13 were on par with the global rate, and the remainder exceeded it.

## Discussion

TS is a rare genetic disorder primarily caused by the deletion or structural abnormalities of the X chromosome, making it one of the typical sex chromosome abnormalities (20). Although the global prevalence of TS is low (approximately 1 in 2,500 female live births (21)), many cases remain undiagnosed due to the subtlety of early symptoms or delayed diagnosis, resulting in a discrepancy between the number of confirmed cases and the actual prevalence (22). Studies have indicated that in countries where Down syndrome screening is routinely performed, the detection rate of TS fetuses is relatively higher (23, 24). This may be attributed to screening technologies that not only identify common chromosomal abnormalities such as Trisomy 21 but also detect other sex chromosome anomalies (25). Additionally, the comprehensive enhancement of screening protocols has led to an increased identification rate of various chromosomal abnormalities. In recent years, the diagnosis rate of TS mosaics has been steadily rising, primarily due to the continuous improvement of screening methods, including the application of high-throughput sequencing and non-invasive prenatal testing (NIPT) technologies (26), as well as an increase in the rate of elective terminations, which partly reflects the rise in early diagnostic interventions. Patients with TS often present with multiple comorbidities, including congenital heart and renal malformations, endocrine dysfunctions, gastrointestinal issues, and psychosocial disorders (27). These comorbidities not only significantly impact the quality of life of patients but also increase the demand for medical resources, thereby imposing a substantial burden on public health systems.

The epidemiological trends of TS from 1990 to 2021 highlight stark disparities between high- and low-SDI regions, driven by population dynamics, healthcare access, and systemic inequities. Globally, TS prevalence cases increased by 12.81%, primarily due to population growth, particularly in the low SDI region, where cases surged by 88.93% alongside rapid demographic expansion. However, declining prevalence rates (e.g., 15.03 to 14.12 per 100,000 in the low SDI region) and stable DALY rates (0.17 per 100,000 globally) suggest improved survival and partial diagnostic advances, albeit overshadowed by persistent management gaps. In high-SDI regions, widespread prenatal screening and multidisciplinary care reduced both prevalence cases (-6.18%) and DALYs (-67.26%), demonstrating the efficacy of early interventions. Conversely, low SDI regions face an interesting phenomenon: improved neonatal survival increases TS cases, but inadequate access to therapy, cardiac monitoring, and psychosocial support exacerbates long-term disability burdens, reflected in rising DALY cases (+1.06%) despite marginally declining rates. Further, accurately estimating the prevalence of TS requires distinguishing clinically diagnosed cases from the true disease burden, as many affected individuals remain undetected. International studies analyzing umbilical cord blood chromosomes in newborns (Denmark, Canada, Japan, etc.) report a biological prevalence of ~64 per 100,000 females (28–30)—a rate nearly double that observed in population-based registries from high-income countries (17–35 per 100,000) (25, 31, 32). This discrepancy likely reflects underdiagnosis due to delayed recognition, early mortality, or atypical presentations. Supporting this, genomic analyses of the UK Biobank cohort (n=245,000) identified numerous undiagnosed mosaic TS cases (45, X/46, XX) through SNP-array profiling (18), demonstrating that standard clinical practices miss subtle or mosaic forms of TS, even in well-resourced settings. These inequities underscore the interplay of population growth, fragmented healthcare systems, and cultural barriers, necessitating global collaboration to prioritize affordable screening tools, subsidized therapies, and integrated TS management within primary healthcare frameworks to bridge the gap between survival and quality of life.

As TS increasingly becomes a significant global public health concern, early diagnosis and intervention are of paramount importance. Early treatments, particularly the use of growth hormone, have been shown to markedly improve patients’ growth and overall prognosis (33). Effective screening programs can enhance the sensitivity and specificity of diagnoses, reduce the time to diagnosis, and facilitate early interventions. This, in turn, alleviates the health burden on patients and their families and optimizes the allocation of medical resources. Therefore, the refinement and widespread implementation of efficient screening technologies are crucial for improving the early diagnostic rates of TS and enhancing patient outcomes.

Despite leveraging the comprehensive GBD database, this study has several inherent limitations. Firstly, data quality and completeness vary across regions and countries within the GBD dataset. In particular, low-income countries often report less accurate or incomplete data, which may compromise the precision of the estimated disease burden for TS in these areas. Secondly, the clinical diagnosis and treatment of TS exhibit significant regional disparities. In low-income settings, the scarcity of diagnostic tools and limited access to healthcare resources can lead to delayed diagnoses or misdiagnoses, thereby affecting the reliability of prevalence and DALYs estimates. Additionally, early comorbidities associated with TS, such as cardiovascular issues or hearing loss, may not be adequately captured in the GBD database. This underreporting can result in an underestimation of both mortality and disability rates related to TS. Furthermore, while this study explores the impact of socioeconomic factors and healthcare accessibility on the disease burden of TS, the measurement of these variables in the GBD database is relatively coarse. The analysis does not account for specific health policies, variations in healthcare systems, or cultural factors within each region, which can significantly influence health outcomes. Lastly, many comorbid conditions associated with TS, including cardiovascular diseases and diabetes, typically manifest in adulthood. Consequently, the burden of these long-term complications is not fully reflected in the assessment of disease burden during childhood (ages 0–14 years). This limitation suggests that the overall impact of TS may be underestimated when focusing solely on the pediatric population. Future research should aim to incorporate more granular data, particularly from low-income regions, and consider longitudinal studies that track the long-term health outcomes of individuals with TS. Additionally, integrating detailed information on healthcare policies and cultural contexts could provide a more nuanced understanding of the factors influencing the disease burden of TS globally.

## Conclusions

Our study thoroughly examines the global and regional burden of pediatric TS from 1990 to 2021. While the total number of TS cases rose worldwide, the overall prevalence and DALY rates changed only slightly, and large differences were observed across various regions and countries. High-SDI regions generally showed higher TS rates and DALYs, while low-SDI regions had the greatest cases. Notably, only the low SDI region experienced an increase in TS-related DALYs, reflecting potential challenges in healthcare access and early detection. Meanwhile, some regions (like Andean Latin America) showed significant decreases in prevalence and DALYs, suggesting that effective healthcare measures can help lower the TS burden. At the national level, India had the most TS cases, while New Zealand showed the highest prevalence and DALY rates, highlighting differences in population size, screening practices, and diagnostic availability. Some countries made progress in reducing the TS burden, but others still face rising cases and DALYs. These findings stress the importance of early intervention, better public health strategies, and sufficient healthcare resources to lessen the global impact of pediatric TS.

## Data Availability

The original contributions presented in the study are included in the article/**Supplementary Material**. Further inquiries can be directed to the corresponding authors.

## Supplementary material

Supplementary Figure 1 Age-Specific Rate of Childhood Turner Syndrome Prevalence and DALYs in 2021. **(A)** Prevalence. **(B)** DALYs.

Supplementary Figure 2 Disability-Adjusted Life Years (DALYs) of Turner Syndrome in Children in 204 Countries and Territories. **(A)** DALYs cases. **(B)** DALYs rate. **(C)** Change in DALYs cases.
