# Corrigendum: Epidemiological analysis of turner syndrome in children aged 0–14 years: global, regional, and national perspectives (1990-2021)

**DOI:** 10.3389/fendo.2025.1620882

**Published:** 2025-05-16

**Authors:** Fang Ding, Jiaoli Xu, Jingxuan Xiong, Qinhong Li, Zugen Cheng, Lili Deng

**Affiliations:** ^1^ Department of Cardiology, Kunming Children’s Hospital, Kunming, China; ^2^ Department of Respiratory Medicine, Kunming Children’s Hospital, Kunming, China; ^3^ Department of Cardiology, The First Affiliated Hospital of Kunming Medical University, Kunming, China

**Keywords:** turner syndrome, GBD (global burden disease), prevalence, DALYs - disability-adjusted life years, children

In the published article, there was an error in the numbering order of the author affiliations. Affiliations 2 and 3 were inverted. The corrected affiliations appear below:

“2. Department of Respiratory Medicine, Kunming Children’s Hospital, Kunming, China

3. Department of Cardiology, The First Affiliated Hospital of Kunming Medical University, Kunming, China”

The authors apologize for this error and state that this does not change the scientific conclusions of the article in any way. The original article has been updated.

