# Supplementary figures and images for: Epidemiological analysis of turner syndrome in children aged 0–14 years: global, regional, and national perspectives (1990-2021)
Source: Front Endocrinol (Lausanne). 2025 Apr 30;16:1552300. doi: 10.3389/fendo.2025.1552300 (PMC12074904; doi:10.3389/fendo.2025.1552300)

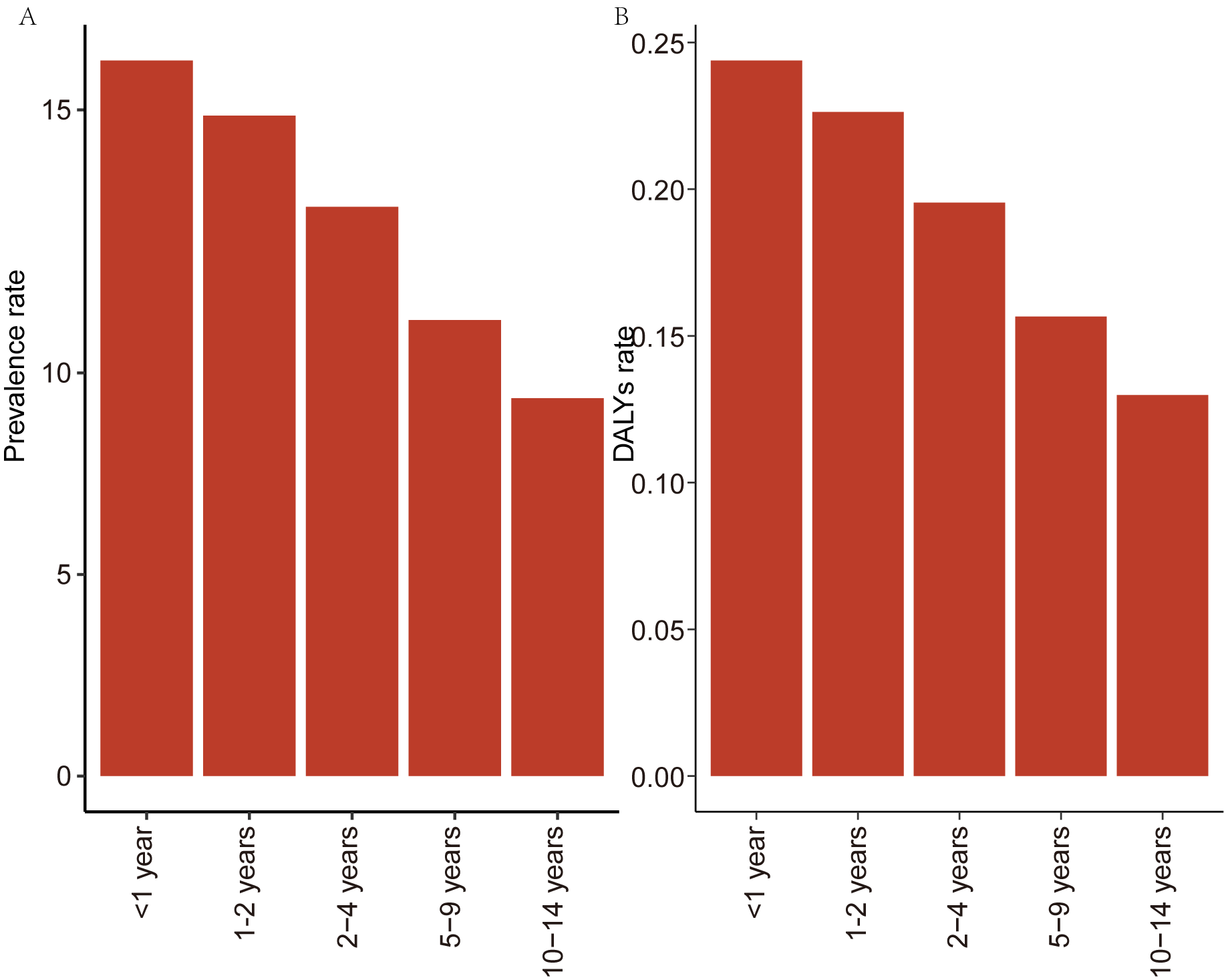

Supplement: Supplementary Figure 1 — Age-Specific Rate of Childhood Turner Syndrome Prevalence and DALYs in 2021. (A) Prevalence. (B) DALYs. [file Image1.tif]

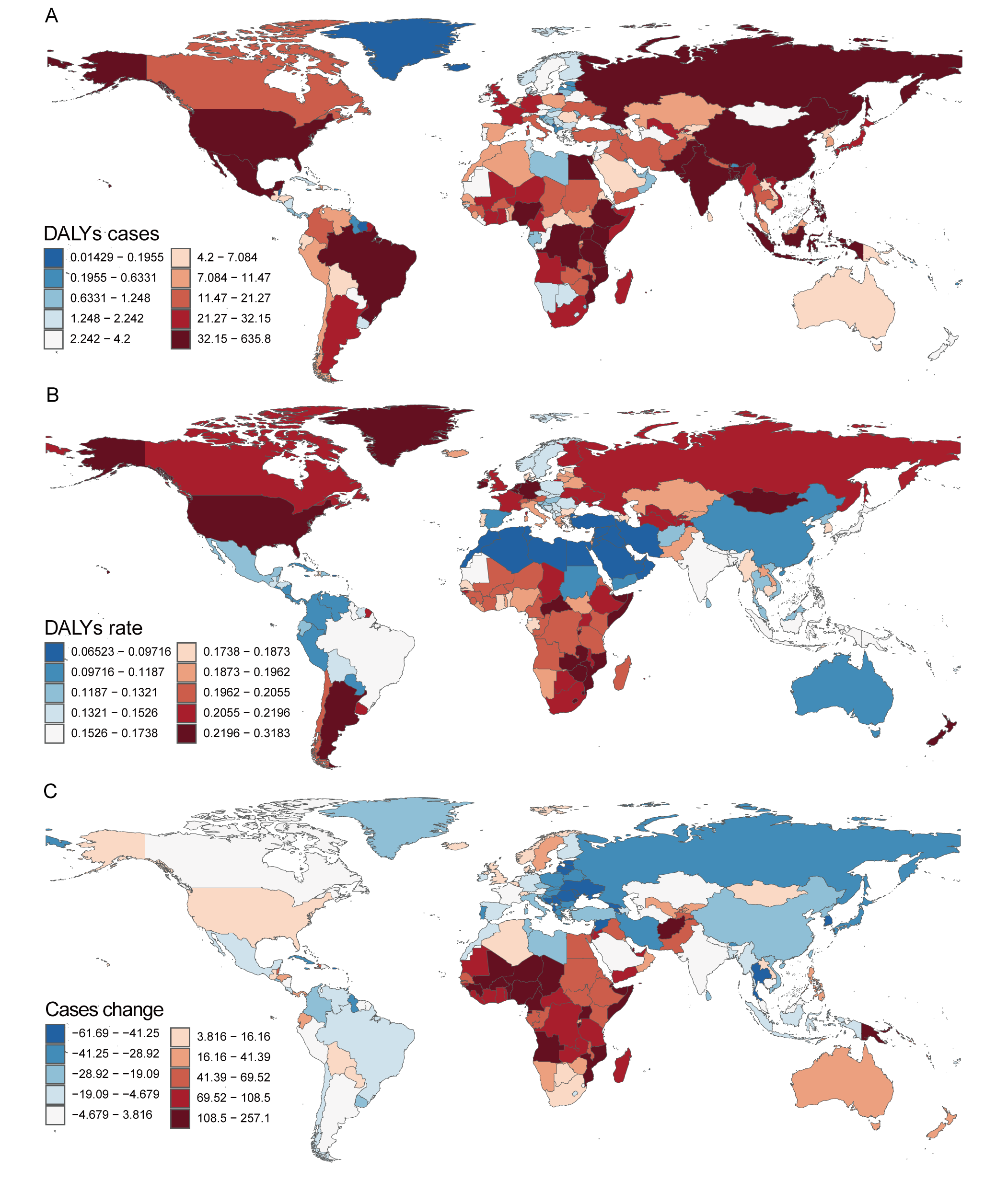

Supplement: Supplementary Figure 2 — Disability-Adjusted Life Years (DALYs) of Turner Syndrome in Children in 204 Countries and Territories. (A) DALYs cases. (B) DALYs rate. (C) Change in DALYs cases. [file Image2.tif]
