# Supplementary material for: Epidemiological analysis of turner syndrome in children aged 0–14 years: global, regional, and national perspectives (1990-2021)
Source: Front Endocrinol (Lausanne). 2025 Apr 30;16:1552300. doi: 10.3389/fendo.2025.1552300 (PMC12074904; doi:10.3389/fendo.2025.1552300)
Supplement: Supplementary file 3 [file Table1.docx]

Table S1 DALYs of Turner Syndrome in Children at the National Level.

| location | 1990 | |  | 2021 | |  | 1990-2021 | |
| --- | --- | --- | --- | --- | --- | --- | --- | --- |
|  | DALYs case | DALYs rate |  | DALYs case | DALYs rate |  | Cases change | EAPC |
| Afghanistan | 5.69(2.26,11.02) | 0.13(0.05,0.26) |  | 17.68(7.18,33.78) | 0.12(0.05,0.24) |  | 210.98(168.32,259.62) | -0.35(-0.42,-0.28) |
| Albania | 1.61(0.65,3.04) | 0.14(0.06,0.27) |  | 0.63(0.26,1.22) | 0.14(0.06,0.27) |  | -60.66(-65.50,-55.53) | -0.07(-0.13,-0.02) |
| Algeria | 10.06(4.05,19.26) | 0.09(0.04,0.18) |  | 11.27(4.50,21.58) | 0.08(0.03,0.16) |  | 12.03(-1.82,27.52) | -0.20(-0.25,-0.14) |
| American Samoa | 0.03(0.01,0.05) | 0.14(0.06,0.27) |  | 0.02(0.01,0.04) | 0.13(0.05,0.25) |  | -28.64(-37.76,-16.96) | -0.18(-0.19,-0.16) |
| Andorra | 0.02(0.01,0.03) | 0.18(0.08,0.34) |  | 0.02(0.01,0.03) | 0.18(0.08,0.34) |  | 6.42(-7.24,22.01) | -0.05(-0.07,-0.02) |
| Angola | 10.31(4.11,20.51) | 0.22(0.09,0.43) |  | 31.11(12.18,60.69) | 0.20(0.08,0.40) |  | 201.73(148.44,268.54) | -0.24(-0.28,-0.20) |
| Antigua and Barbuda | 0.02(0.01,0.04) | 0.12(0.05,0.23) |  | 0.02(0.01,0.04) | 0.11(0.04,0.21) |  | -13.20(-24.46,-2.34) | -0.28(-0.31,-0.25) |
| Argentina | 23.28(9.12,45.13) | 0.23(0.09,0.45) |  | 22.71(9.08,42.98) | 0.22(0.09,0.42) |  | -2.45(-15.16,13.05) | -0.05(-0.07,-0.03) |
| Armenia | 1.71(0.68,3.25) | 0.16(0.07,0.31) |  | 0.92(0.36,1.75) | 0.16(0.06,0.30) |  | -46.01(-53.48,-37.81) | -0.14(-0.19,-0.08) |
| Australia | 4.43(1.81,8.13) | 0.12(0.05,0.21) |  | 5.39(2.24,10.24) | 0.11(0.05,0.22) |  | 21.55(6.98,37.32) | -0.07(-0.11,-0.04) |
| Austria | 2.78(1.17,5.08) | 0.21(0.09,0.38) |  | 2.66(1.09,4.97) | 0.21(0.08,0.38) |  | -4.31(-15.35,9.49) | -0.05(-0.08,-0.02) |
| Azerbaijan | 4.65(1.88,8.79) | 0.19(0.08,0.36) |  | 4.28(1.74,7.93) | 0.18(0.07,0.34) |  | -8.08(-19.86,4.94) | -0.17(-0.22,-0.13) |
| Bahamas | 0.11(0.04,0.20) | 0.14(0.05,0.25) |  | 0.10(0.04,0.19) | 0.13(0.05,0.24) |  | -5.84(-17.90,7.92) | -0.23(-0.27,-0.18) |
| Bahrain | 0.14(0.05,0.26) | 0.08(0.03,0.16) |  | 0.22(0.09,0.42) | 0.07(0.03,0.14) |  | 63.87(41.68,82.95) | -0.36(-0.40,-0.33) |
| Bangladesh | 90.10(35.87,173.98) | 0.18(0.07,0.36) |  | 73.06(30.24,140.76) | 0.16(0.07,0.31) |  | -18.91(-30.40,-7.76) | -0.33(-0.37,-0.29) |
| Barbados | 0.08(0.03,0.14) | 0.12(0.05,0.23) |  | 0.05(0.02,0.10) | 0.11(0.04,0.21) |  | -29.62(-38.29,-20.13) | -0.24(-0.25,-0.22) |
| Belarus | 4.90(2.01,9.22) | 0.20(0.08,0.38) |  | 3.07(1.26,5.66) | 0.19(0.08,0.36) |  | -37.46(-45.45,-28.40) | -0.04(-0.09,0.00) |
| Belgium | 5.05(1.92,9.85) | 0.28(0.11,0.55) |  | 5.25(2.18,9.86) | 0.27(0.11,0.52) |  | 4.09(-25.54,44.89) | -0.23(-0.27,-0.18) |
| Belize | 0.11(0.04,0.22) | 0.14(0.05,0.26) |  | 0.16(0.06,0.30) | 0.13(0.05,0.24) |  | 42.31(26.16,62.22) | -0.18(-0.20,-0.17) |
| Benin | 4.65(1.88,8.94) | 0.19(0.08,0.37) |  | 11.32(4.44,22.42) | 0.19(0.07,0.37) |  | 143.37(108.80,181.79) | -0.08(-0.10,-0.06) |
| Bermuda | 0.01(0.00,0.02) | 0.10(0.04,0.19) |  | 0.01(0.00,0.01) | 0.09(0.04,0.16) |  | -38.56(-45.94,-30.08) | -0.44(-0.47,-0.42) |
| Bhutan | 0.45(0.18,0.84) | 0.17(0.07,0.32) |  | 0.29(0.12,0.54) | 0.16(0.07,0.29) |  | -34.22(-42.42,-25.46) | -0.23(-0.25,-0.20) |
| Bolivia (Plurinational State of) | 4.27(1.71,7.98) | 0.16(0.06,0.30) |  | 4.96(1.98,9.67) | 0.14(0.06,0.28) |  | 16.16(2.51,32.86) | -0.33(-0.36,-0.29) |
| Bosnia and Herzegovina | 1.53(0.63,2.83) | 0.14(0.06,0.26) |  | 0.70(0.28,1.27) | 0.14(0.06,0.26) |  | -54.51(-60.34,-48.50) | 0.02(-0.03,0.07) |
| Botswana | 1.32(0.50,2.66) | 0.22(0.09,0.45) |  | 1.46(0.61,2.79) | 0.21(0.09,0.40) |  | 10.36(-6.86,30.57) | -0.23(-0.26,-0.20) |
| Brazil | 87.59(36.20,164.69) | 0.17(0.07,0.32) |  | 81.21(34.05,152.19) | 0.17(0.07,0.32) |  | -7.29(-12.99,-1.89) | 0.02(0.01,0.03) |
| Brunei Darussalam | 0.21(0.09,0.39) | 0.23(0.09,0.43) |  | 0.21(0.09,0.41) | 0.23(0.09,0.43) |  | 2.11(-11.33,18.35) | -0.13(-0.17,-0.09) |
| Bulgaria | 3.07(1.22,5.87) | 0.18(0.07,0.34) |  | 1.81(0.72,3.40) | 0.19(0.07,0.35) |  | -41.11(-47.79,-32.41) | 0.11(0.02,0.19) |
| Burkina Faso | 9.84(3.77,19.09) | 0.21(0.08,0.40) |  | 21.27(8.52,41.74) | 0.21(0.08,0.40) |  | 116.11(78.16,162.39) | -0.03(-0.05,-0.01) |
| Burundi | 6.10(2.27,11.57) | 0.23(0.09,0.44) |  | 12.90(4.92,24.89) | 0.22(0.08,0.43) |  | 111.31(56.37,176.83) | -0.12(-0.15,-0.09) |
| Cabo Verde | 0.23(0.09,0.44) | 0.15(0.06,0.28) |  | 0.19(0.08,0.36) | 0.13(0.05,0.25) |  | -17.31(-27.46,-5.97) | -0.30(-0.33,-0.28) |
| Cambodia | 9.55(3.81,18.38) | 0.20(0.08,0.39) |  | 9.12(3.82,17.22) | 0.18(0.07,0.34) |  | -4.53(-18.94,12.15) | -0.46(-0.52,-0.39) |
| Cameroon | 10.25(4.03,20.33) | 0.21(0.08,0.42) |  | 26.79(10.64,51.81) | 0.20(0.08,0.38) |  | 161.30(115.64,210.54) | -0.13(-0.17,-0.09) |
| Canada | 12.55(5.23,23.48) | 0.22(0.09,0.41) |  | 12.97(5.41,24.46) | 0.21(0.09,0.40) |  | 3.36(-11.56,22.10) | -0.10(-0.12,-0.09) |
| Central African Republic | 3.12(1.10,7.00) | 0.26(0.09,0.57) |  | 5.53(1.76,11.92) | 0.24(0.08,0.52) |  | 76.94(-5.59,236.44) | -0.11(-0.15,-0.08) |
| Chad | 6.43(2.53,12.83) | 0.22(0.09,0.44) |  | 18.94(7.56,36.12) | 0.21(0.08,0.40) |  | 194.32(132.49,261.17) | -0.12(-0.16,-0.09) |
| Chile | 8.40(3.49,15.93) | 0.21(0.09,0.40) |  | 7.29(2.95,13.65) | 0.20(0.08,0.37) |  | -13.31(-24.33,0.28) | -0.18(-0.20,-0.15) |
| China | 334.27(138.13,631.19) | 0.10(0.04,0.20) |  | 258.37(105.26,486.92) | 0.10(0.04,0.19) |  | -22.71(-26.02,-18.70) | -0.15(-0.22,-0.09) |
| Colombia | 14.57(6.01,27.38) | 0.12(0.05,0.23) |  | 11.63(4.66,22.38) | 0.11(0.04,0.21) |  | -20.19(-30.15,-8.87) | -0.41(-0.44,-0.38) |
| Comoros | 0.40(0.17,0.78) | 0.19(0.08,0.37) |  | 0.43(0.17,0.81) | 0.18(0.07,0.34) |  | 5.45(-7.31,20.91) | -0.29(-0.32,-0.27) |
| Congo | 2.36(0.89,4.43) | 0.22(0.08,0.42) |  | 3.95(1.57,7.61) | 0.20(0.08,0.39) |  | 67.43(35.55,105.04) | -0.35(-0.40,-0.30) |
| Cook Islands | 0.01(0.00,0.02) | 0.12(0.05,0.23) |  | 0.00(0.00,0.01) | 0.11(0.05,0.21) |  | -46.46(-54.31,-37.82) | -0.22(-0.27,-0.17) |
| Costa Rica | 1.20(0.49,2.29) | 0.11(0.04,0.20) |  | 1.02(0.42,1.93) | 0.10(0.04,0.19) |  | -14.96(-25.14,-2.01) | -0.15(-0.17,-0.14) |
| Croatia | 12.15(4.82,23.83) | 0.21(0.08,0.42) |  | 22.71(9.11,43.21) | 0.20(0.08,0.37) |  | -38.69(-47.07,-30.87) | -0.20(-0.23,-0.18) |
| Cuba | 1.28(0.52,2.33) | 0.13(0.05,0.24) |  | 0.79(0.32,1.47) | 0.13(0.05,0.25) |  | -33.76(-42.49,-24.54) | 0.04(0.02,0.06) |
| Cyprus | 2.79(1.14,5.19) | 0.11(0.05,0.21) |  | 1.85(0.74,3.43) | 0.10(0.04,0.19) |  | 4.65(-7.63,17.80) | -0.18(-0.22,-0.14) |
| Czechia | 0.40(0.16,0.73) | 0.20(0.08,0.37) |  | 0.41(0.17,0.77) | 0.19(0.08,0.35) |  | -21.63(-30.18,-11.02) | -0.18(-0.20,-0.17) |
| C么te d'Ivoire | 3.34(1.32,6.19) | 0.15(0.06,0.28) |  | 2.62(1.07,4.84) | 0.15(0.06,0.28) |  | 86.90(55.88,121.43) | 0.06(-0.01,0.14) |
| Democratic People's Republic of Korea | 7.51(3.09,14.27) | 0.13(0.05,0.24) |  | 5.75(2.28,10.74) | 0.12(0.05,0.23) |  | -23.45(-33.13,-11.57) | -0.13(-0.16,-0.11) |
| Democratic Republic of the Congo | 37.86(14.80,73.77) | 0.21(0.08,0.42) |  | 75.61(28.32,145.56) | 0.20(0.07,0.38) |  | 99.69(64.00,144.85) | -0.19(-0.23,-0.16) |
| Denmark | 1.58(0.63,2.89) | 0.18(0.07,0.33) |  | 1.68(0.70,3.23) | 0.18(0.07,0.34) |  | 6.48(-8.12,23.44) | 0.03(-0.01,0.06) |
| Djibouti | 0.32(0.13,0.62) | 0.19(0.08,0.35) |  | 0.71(0.29,1.37) | 0.17(0.07,0.33) |  | 121.45(97.88,153.76) | -0.26(-0.28,-0.24) |
| Dominica | 0.03(0.01,0.06) | 0.13(0.05,0.25) |  | 0.02(0.01,0.03) | 0.13(0.05,0.24) |  | -47.04(-53.92,-39.32) | -0.16(-0.18,-0.14) |
| Dominican Republic | 3.80(1.61,7.35) | 0.14(0.06,0.27) |  | 3.83(1.66,7.18) | 0.13(0.06,0.24) |  | 0.94(-12.90,15.54) | -0.25(-0.31,-0.18) |
| Ecuador | 5.24(2.11,10.00) | 0.14(0.05,0.26) |  | 6.20(2.59,11.78) | 0.12(0.05,0.23) |  | 18.35(1.98,37.73) | -0.31(-0.34,-0.29) |
| Egypt | 22.25(8.89,43.43) | 0.10(0.04,0.20) |  | 33.81(13.64,65.69) | 0.09(0.04,0.18) |  | 51.97(35.29,76.55) | -0.16(-0.20,-0.11) |
| El Salvador | 2.91(1.21,5.52) | 0.13(0.06,0.26) |  | 2.16(0.83,4.19) | 0.12(0.05,0.23) |  | -25.81(-35.99,-14.53) | -0.43(-0.49,-0.37) |
| Equatorial Guinea | 0.44(0.17,0.84) | 0.22(0.09,0.43) |  | 1.01(0.41,1.94) | 0.17(0.07,0.33) |  | 132.07(83.27,194.17) | -0.93(-1.00,-0.86) |
| Eritrea | 3.33(1.34,6.67) | 0.21(0.08,0.42) |  | 5.06(2.04,9.65) | 0.20(0.08,0.38) |  | 51.99(23.75,86.24) | -0.14(-0.15,-0.12) |
| Estonia | 0.68(0.27,1.28) | 0.19(0.08,0.37) |  | 0.40(0.16,0.75) | 0.18(0.07,0.35) |  | -41.39(-49.57,-34.20) | -0.15(-0.20,-0.11) |
| Eswatini | 0.91(0.35,1.78) | 0.24(0.09,0.46) |  | 0.91(0.35,1.80) | 0.22(0.08,0.44) |  | -0.19(-21.36,31.68) | -0.23(-0.29,-0.17) |
| Ethiopia | 55.14(21.91,102.87) | 0.23(0.09,0.42) |  | 92.78(37.16,173.91) | 0.21(0.08,0.39) |  | 68.28(48.06,90.97) | -0.35(-0.39,-0.32) |
| Fiji | 0.43(0.18,0.83) | 0.15(0.06,0.29) |  | 0.41(0.17,0.79) | 0.15(0.06,0.29) |  | -4.78(-17.17,7.25) | -0.01(-0.03,0.02) |
| Finland | 2.20(0.92,4.04) | 0.23(0.10,0.42) |  | 1.85(0.77,3.40) | 0.22(0.09,0.40) |  | -15.72(-25.54,-4.32) | -0.10(-0.12,-0.09) |
| France | 23.52(9.79,44.40) | 0.20(0.08,0.38) |  | 24.36(10.20,44.54) | 0.21(0.09,0.38) |  | 3.54(-13.82,25.60) | 0.20(-0.06,0.45) |
| Gabon | 0.81(0.33,1.56) | 0.20(0.08,0.38) |  | 1.18(0.49,2.21) | 0.18(0.08,0.35) |  | 46.31(27.18,69.20) | -0.26(-0.30,-0.22) |
| Gambia | 0.90(0.37,1.72) | 0.19(0.08,0.37) |  | 1.82(0.74,3.53) | 0.18(0.07,0.36) |  | 102.03(74.42,131.18) | -0.18(-0.21,-0.16) |
| Georgia | 2.30(0.93,4.20) | 0.17(0.07,0.31) |  | 1.26(0.51,2.36) | 0.17(0.07,0.32) |  | -45.21(-51.65,-37.01) | 0.14(0.08,0.21) |
| Germany | 33.45(13.27,63.60) | 0.26(0.10,0.49) |  | 30.35(12.44,55.07) | 0.25(0.10,0.46) |  | -9.26(-21.93,4.24) | -0.12(-0.17,-0.08) |
| Ghana | 12.96(5.43,24.29) | 0.19(0.08,0.36) |  | 23.59(9.86,44.32) | 0.18(0.08,0.34) |  | 81.95(60.65,107.61) | -0.13(-0.16,-0.11) |
| Greece | 3.81(1.58,7.03) | 0.19(0.08,0.35) |  | 2.68(1.13,4.91) | 0.19(0.08,0.35) |  | -29.68(-38.16,-20.46) | 0.08(0.06,0.10) |
| Greenland | 0.05(0.01,0.10) | 0.33(0.10,0.69) |  | 0.04(0.01,0.09) | 0.32(0.09,0.75) |  | -20.00(-64.01,75.80) | -0.17(-0.21,-0.12) |
| Grenada | 0.05(0.02,0.09) | 0.14(0.05,0.26) |  | 0.03(0.01,0.05) | 0.12(0.05,0.24) |  | -40.27(-47.60,-31.75) | -0.32(-0.34,-0.29) |
| Guam | 0.05(0.02,0.10) | 0.13(0.05,0.25) |  | 0.05(0.02,0.09) | 0.12(0.05,0.23) |  | -15.23(-27.27,-3.13) | -0.15(-0.22,-0.08) |
| Guatemala | 6.64(2.63,12.95) | 0.16(0.06,0.32) |  | 7.08(2.93,13.82) | 0.14(0.06,0.28) |  | 6.73(-6.72,21.01) | -0.39(-0.43,-0.34) |
| Guinea | 5.87(2.30,11.61) | 0.21(0.08,0.42) |  | 12.33(4.95,23.99) | 0.20(0.08,0.40) |  | 110.08(72.16,161.72) | -0.15(-0.17,-0.14) |
| Guinea-Bissau | 1.10(0.42,2.13) | 0.23(0.09,0.44) |  | 1.94(0.71,3.73) | 0.22(0.08,0.42) |  | 76.62(40.92,128.14) | -0.13(-0.17,-0.09) |
| Guyana | 0.49(0.19,0.97) | 0.17(0.07,0.33) |  | 0.33(0.14,0.63) | 0.16(0.06,0.30) |  | -32.56(-40.96,-21.63) | -0.26(-0.32,-0.20) |
| Haiti | 5.48(2.15,10.40) | 0.20(0.08,0.38) |  | 8.07(3.19,16.03) | 0.19(0.07,0.37) |  | 47.43(28.35,70.76) | -0.21(-0.24,-0.17) |
| Honduras | 3.39(1.39,6.51) | 0.15(0.06,0.29) |  | 4.66(1.88,9.03) | 0.14(0.06,0.28) |  | 37.52(19.20,53.20) | -0.25(-0.27,-0.24) |
| Hungary | 2.62(1.03,4.99) | 0.12(0.05,0.23) |  | 1.73(0.71,3.11) | 0.12(0.05,0.22) |  | -34.09(-41.94,-24.10) | 0.04(-0.05,0.13) |
| Iceland | 0.12(0.05,0.22) | 0.19(0.08,0.35) |  | 0.13(0.05,0.24) | 0.19(0.08,0.36) |  | 7.03(-5.21,22.18) | 0.03(0.02,0.04) |
| India | 625.99(252.42,1200.85) | 0.19(0.08,0.37) |  | 635.79(255.73,1220.19) | 0.17(0.07,0.33) |  | 1.56(-3.43,6.29) | -0.28(-0.30,-0.26) |
| Indonesia | 124.70(50.99,236.81) | 0.18(0.08,0.35) |  | 112.85(45.43,214.15) | 0.17(0.07,0.32) |  | -9.51(-12.71,-5.95) | -0.34(-0.37,-0.32) |
| Iran (Islamic Republic of) | 23.22(9.39,44.08) | 0.09(0.04,0.17) |  | 16.50(6.75,31.29) | 0.08(0.03,0.16) |  | -28.92(-31.40,-25.94) | -0.14(-0.20,-0.08) |
| Iraq | 8.02(3.17,15.33) | 0.10(0.04,0.19) |  | 11.88(4.76,23.02) | 0.09(0.04,0.17) |  | 48.17(31.53,68.56) | -0.35(-0.38,-0.32) |
| Ireland | 2.60(1.00,4.96) | 0.26(0.10,0.50) |  | 2.48(1.03,4.60) | 0.25(0.10,0.46) |  | -4.68(-21.65,14.25) | -0.06(-0.10,-0.03) |
| Israel | 3.00(1.23,5.49) | 0.20(0.08,0.36) |  | 5.16(2.11,9.57) | 0.20(0.08,0.36) |  | 71.82(51.44,97.11) | -0.01(-0.03,0.00) |
| Italy | 18.10(7.53,32.95) | 0.20(0.08,0.36) |  | 14.53(6.02,26.75) | 0.19(0.08,0.35) |  | -19.73(-22.24,-16.82) | -0.10(-0.11,-0.09) |
| Jamaica | 1.02(0.41,1.87) | 0.12(0.05,0.22) |  | 0.67(0.27,1.28) | 0.12(0.05,0.22) |  | -34.37(-42.47,-25.01) | -0.20(-0.25,-0.15) |
| Japan | 40.54(16.74,73.43) | 0.18(0.07,0.32) |  | 26.38(11.03,48.32) | 0.17(0.07,0.31) |  | -34.94(-36.81,-32.95) | -0.07(-0.09,-0.05) |
| Jordan | 1.38(0.55,2.59) | 0.08(0.03,0.16) |  | 2.80(1.17,5.18) | 0.08(0.03,0.14) |  | 102.60(77.83,134.62) | -0.36(-0.39,-0.33) |
| Kazakhstan | 10.26(4.13,19.61) | 0.20(0.08,0.38) |  | 10.56(4.27,20.28) | 0.19(0.08,0.37) |  | 2.85(-10.94,18.47) | -0.05(-0.08,-0.02) |
| Kenya | 23.41(9.46,44.43) | 0.21(0.08,0.40) |  | 37.42(15.24,71.67) | 0.20(0.08,0.38) |  | 59.86(53.91,66.05) | -0.10(-0.15,-0.06) |
| Kiribati | 0.06(0.02,0.11) | 0.19(0.08,0.36) |  | 0.07(0.03,0.14) | 0.18(0.07,0.33) |  | 32.72(15.27,54.02) | -0.15(-0.20,-0.11) |
| Kuwait | 0.39(0.16,0.73) | 0.07(0.03,0.13) |  | 0.55(0.22,1.03) | 0.07(0.03,0.12) |  | 40.04(23.01,59.68) | -0.24(-0.25,-0.22) |
| Kyrgyzstan | 3.32(1.32,6.37) | 0.20(0.08,0.38) |  | 4.46(1.79,8.40) | 0.20(0.08,0.37) |  | 34.20(17.11,56.07) | 0.02(-0.02,0.07) |
| Lao People's Democratic Republic | 3.93(1.54,7.66) | 0.21(0.08,0.42) |  | 4.40(1.83,8.30) | 0.19(0.08,0.36) |  | 11.89(-12.04,39.48) | -0.43(-0.46,-0.40) |
| Latvia | 1.16(0.46,2.21) | 0.20(0.08,0.39) |  | 0.58(0.23,1.06) | 0.20(0.08,0.36) |  | -50.06(-57.18,-42.65) | -0.01(-0.07,0.04) |
| Lebanon | 0.88(0.35,1.67) | 0.08(0.03,0.16) |  | 0.96(0.38,1.79) | 0.08(0.03,0.14) |  | 9.00(-5.51,22.65) | -0.34(-0.37,-0.32) |
| Lesotho | 1.63(0.60,3.16) | 0.24(0.09,0.46) |  | 1.49(0.56,2.91) | 0.24(0.09,0.46) |  | -9.03(-29.97,17.79) | -0.02(-0.05,0.01) |
| Liberia | 2.38(0.93,4.58) | 0.21(0.08,0.41) |  | 4.12(1.71,8.18) | 0.19(0.08,0.37) |  | 73.20(48.04,102.67) | -0.32(-0.36,-0.28) |
| Libya | 1.63(0.67,3.12) | 0.09(0.04,0.17) |  | 1.19(0.49,2.31) | 0.08(0.03,0.15) |  | -26.95(-35.10,-18.31) | -0.45(-0.49,-0.42) |
| Lithuania | 1.66(0.66,3.14) | 0.20(0.08,0.38) |  | 0.80(0.33,1.54) | 0.20(0.08,0.38) |  | -51.73(-57.60,-44.43) | 0.04(-0.04,0.12) |
| Luxembourg | 0.14(0.06,0.25) | 0.21(0.08,0.39) |  | 0.20(0.08,0.37) | 0.20(0.08,0.36) |  | 45.60(29.01,67.11) | -0.18(-0.21,-0.16) |
| Madagascar | 11.33(4.53,21.43) | 0.21(0.08,0.39) |  | 23.44(9.62,45.18) | 0.20(0.08,0.39) |  | 106.90(73.32,153.89) | -0.15(-0.17,-0.13) |
| Malawi | 10.99(4.11,22.08) | 0.24(0.09,0.49) |  | 17.75(6.65,35.34) | 0.22(0.08,0.43) |  | 61.56(19.44,115.15) | -0.30(-0.36,-0.25) |
| Malaysia | 9.51(3.73,18.49) | 0.14(0.06,0.28) |  | 9.73(3.91,18.19) | 0.13(0.05,0.24) |  | 2.35(-8.19,17.02) | -0.45(-0.50,-0.40) |
| Maldives | 0.17(0.07,0.33) | 0.16(0.06,0.31) |  | 0.13(0.05,0.25) | 0.13(0.05,0.25) |  | -23.67(-34.38,-12.30) | -0.70(-0.80,-0.61) |
| Mali | 8.87(3.36,17.17) | 0.21(0.08,0.42) |  | 23.60(8.93,46.11) | 0.20(0.08,0.40) |  | 166.18(111.67,230.56) | -0.15(-0.17,-0.13) |
| Malta | 0.17(0.07,0.32) | 0.19(0.08,0.36) |  | 0.12(0.05,0.23) | 0.19(0.08,0.36) |  | -27.55(-37.19,-17.53) | -0.01(-0.02,0.01) |
| Marshall Islands | 0.04(0.01,0.07) | 0.17(0.07,0.32) |  | 0.03(0.01,0.06) | 0.17(0.07,0.32) |  | -23.01(-32.63,-10.80) | -0.09(-0.12,-0.05) |
| Mauritania | 1.75(0.71,3.36) | 0.19(0.08,0.36) |  | 3.17(1.26,6.07) | 0.17(0.07,0.33) |  | 81.30(57.69,107.35) | -0.27(-0.31,-0.24) |
| Mauritius | 0.47(0.19,0.91) | 0.14(0.06,0.27) |  | 0.27(0.11,0.51) | 0.13(0.05,0.25) |  | -41.74(-48.75,-33.12) | -0.31(-0.35,-0.27) |
| Mexico | 45.06(18.29,85.12) | 0.13(0.05,0.25) |  | 39.21(15.93,73.58) | 0.12(0.05,0.23) |  | -12.97(-16.00,-9.83) | -0.25(-0.29,-0.22) |
| Micronesia (Federated States of) | 0.08(0.03,0.15) | 0.17(0.07,0.32) |  | 0.05(0.02,0.10) | 0.16(0.07,0.31) |  | -35.77(-43.15,-26.97) | -0.14(-0.16,-0.13) |
| Monaco | 0.01(0.00,0.01) | 0.19(0.08,0.36) |  | 0.01(0.00,0.02) | 0.21(0.09,0.39) |  | 50.70(33.27,72.48) | 0.19(0.17,0.20) |
| Mongolia | 2.13(0.84,4.14) | 0.24(0.09,0.46) |  | 2.43(0.93,4.59) | 0.22(0.09,0.42) |  | 14.21(-14.27,50.50) | -0.10(-0.16,-0.04) |
| Montenegro | 0.22(0.09,0.40) | 0.14(0.05,0.25) |  | 0.16(0.06,0.29) | 0.14(0.06,0.26) |  | -28.06(-36.50,-18.71) | 0.09(0.07,0.11) |
| Morocco | 9.77(4.17,18.62) | 0.10(0.04,0.19) |  | 8.77(3.49,16.78) | 0.09(0.04,0.17) |  | -10.26(-20.92,3.77) | -0.26(-0.29,-0.23) |
| Mozambique | 14.61(5.83,28.74) | 0.24(0.09,0.46) |  | 32.44(12.25,64.34) | 0.23(0.09,0.45) |  | 122.09(65.61,198.68) | -0.06(-0.10,-0.02) |
| Myanmar | 29.41(11.46,55.42) | 0.20(0.08,0.38) |  | 27.48(11.17,52.01) | 0.18(0.07,0.33) |  | -6.55(-20.67,10.88) | -0.47(-0.49,-0.45) |
| Namibia | 1.24(0.49,2.33) | 0.21(0.08,0.39) |  | 1.59(0.65,3.06) | 0.19(0.08,0.37) |  | 28.06(12.60,46.79) | -0.23(-0.26,-0.21) |
| Nauru | 0.01(0.00,0.01) | 0.17(0.07,0.34) |  | 0.01(0.00,0.01) | 0.17(0.07,0.32) |  | -10.27(-20.59,2.12) | -0.17(-0.22,-0.12) |
| Nepal | 15.35(6.06,29.51) | 0.18(0.07,0.35) |  | 15.12(6.34,28.93) | 0.16(0.07,0.31) |  | -1.50(-14.01,13.81) | -0.30(-0.33,-0.28) |
| Netherlands | 6.47(2.71,11.98) | 0.24(0.10,0.44) |  | 6.30(2.63,11.54) | 0.23(0.10,0.43) |  | -2.61(-15.47,10.02) | -0.01(-0.09,0.07) |
| New Zealand | 2.53(0.95,4.76) | 0.32(0.12,0.59) |  | 3.12(1.21,6.20) | 0.32(0.12,0.63) |  | 23.64(-15.48,71.58) | 0.05(0.02,0.07) |
| Nicaragua | 2.28(0.91,4.28) | 0.13(0.05,0.23) |  | 2.24(0.91,4.23) | 0.11(0.05,0.21) |  | -1.79(-13.63,12.87) | -0.32(-0.35,-0.29) |
| Niger | 8.53(3.26,16.24) | 0.21(0.08,0.40) |  | 25.83(10.01,50.30) | 0.20(0.08,0.39) |  | 202.93(144.68,265.25) | -0.18(-0.20,-0.16) |
| Nigeria | 82.16(33.86,152.53) | 0.21(0.09,0.39) |  | 198.65(80.04,378.98) | 0.20(0.08,0.37) |  | 141.79(130.51,152.56) | -0.17(-0.20,-0.14) |
| Niue | 0.00(0.00,0.00) | 0.14(0.05,0.26) |  | 0.00(0.00,0.00) | 0.13(0.05,0.24) |  | -54.54(-60.19,-46.32) | -0.14(-0.23,-0.06) |
| North Macedonia | 0.77(0.31,1.46) | 0.15(0.06,0.28) |  | 0.48(0.19,0.90) | 0.15(0.06,0.28) |  | -37.29(-44.20,-28.19) | 0.02(-0.02,0.05) |
| Northern Mariana Islands | 0.02(0.01,0.03) | 0.13(0.05,0.24) |  | 0.01(0.01,0.03) | 0.12(0.05,0.23) |  | -14.96(-27.08,-1.96) | -0.23(-0.28,-0.18) |
| Norway | 1.20(0.50,2.23) | 0.15(0.06,0.28) |  | 1.39(0.58,2.56) | 0.15(0.06,0.28) |  | 15.61(11.10,20.85) | 0.11(0.06,0.17) |
| Oman | 0.76(0.31,1.45) | 0.09(0.04,0.17) |  | 1.00(0.40,1.91) | 0.08(0.03,0.16) |  | 31.02(15.37,50.77) | -0.23(-0.28,-0.19) |
| Pakistan | 96.76(38.76,194.14) | 0.20(0.08,0.39) |  | 162.17(65.73,314.86) | 0.19(0.08,0.37) |  | 67.60(48.39,86.62) | -0.08(-0.11,-0.05) |
| Palau | 0.01(0.00,0.01) | 0.14(0.06,0.28) |  | 0.00(0.00,0.01) | 0.14(0.06,0.26) |  | -32.34(-41.09,-21.79) | -0.21(-0.24,-0.18) |
| Palestine | 0.82(0.33,1.57) | 0.09(0.03,0.16) |  | 1.47(0.58,2.77) | 0.08(0.03,0.15) |  | 78.60(58.33,102.14) | -0.23(-0.24,-0.21) |
| Panama | 0.98(0.39,1.87) | 0.12(0.05,0.22) |  | 1.24(0.49,2.33) | 0.11(0.04,0.20) |  | 26.62(11.04,42.30) | -0.22(-0.25,-0.20) |
| Papua New Guinea | 2.85(1.15,5.45) | 0.17(0.07,0.32) |  | 6.53(2.52,12.63) | 0.17(0.06,0.32) |  | 128.89(97.84,169.23) | 0.00(-0.02,0.02) |
| Paraguay | 2.11(0.89,3.93) | 0.13(0.05,0.24) |  | 2.35(0.94,4.43) | 0.12(0.05,0.22) |  | 11.44(-3.07,25.17) | -0.20(-0.22,-0.18) |
| Peru | 11.16(4.55,21.49) | 0.13(0.05,0.26) |  | 10.94(4.34,20.90) | 0.11(0.05,0.22) |  | -2.01(-15.80,13.85) | -0.59(-0.61,-0.56) |
| Philippines | 42.91(17.44,81.34) | 0.17(0.07,0.32) |  | 53.74(21.88,100.92) | 0.16(0.06,0.30) |  | 25.24(22.65,28.45) | -0.28(-0.30,-0.26) |
| Poland | 13.98(5.71,25.88) | 0.15(0.06,0.27) |  | 8.72(3.60,16.30) | 0.15(0.06,0.28) |  | -37.66(-40.20,-34.90) | -0.01(-0.15,0.13) |
| Portugal | 3.95(1.54,7.27) | 0.19(0.07,0.34) |  | 2.39(0.95,4.39) | 0.18(0.07,0.32) |  | -39.40(-46.70,-32.24) | -0.21(-0.25,-0.17) |
| Puerto Rico | 1.05(0.43,1.94) | 0.10(0.04,0.20) |  | 0.43(0.17,0.81) | 0.10(0.04,0.18) |  | -59.31(-64.08,-53.52) | -0.33(-0.34,-0.31) |
| Qatar | 0.10(0.04,0.19) | 0.08(0.03,0.16) |  | 0.37(0.15,0.70) | 0.07(0.03,0.14) |  | 257.07(214.03,315.98) | -0.29(-0.32,-0.25) |
| Republic of Korea | 22.56(9.06,41.93) | 0.20(0.08,0.37) |  | 10.82(4.38,19.82) | 0.18(0.07,0.33) |  | -52.03(-58.42,-44.37) | -0.36(-0.40,-0.32) |
| Republic of Moldova | 2.71(1.07,5.12) | 0.22(0.09,0.41) |  | 1.10(0.44,2.06) | 0.21(0.08,0.40) |  | -59.31(-64.79,-53.78) | 0.01(-0.05,0.08) |
| Romania | 8.41(3.23,15.84) | 0.15(0.06,0.28) |  | 4.56(1.86,8.52) | 0.15(0.06,0.28) |  | -45.77(-52.31,-37.88) | 0.04(0.01,0.06) |
| Russian Federation | 80.45(32.75,150.01) | 0.23(0.09,0.43) |  | 56.92(23.20,106.52) | 0.22(0.09,0.41) |  | -29.24(-31.08,-27.29) | -0.05(-0.10,-0.00) |
| Rwanda | 7.45(3.00,14.49) | 0.22(0.09,0.43) |  | 9.83(3.91,19.35) | 0.20(0.08,0.39) |  | 31.84(11.85,58.29) | -0.39(-0.43,-0.35) |
| Saint Kitts and Nevis | 0.02(0.01,0.04) | 0.14(0.06,0.26) |  | 0.01(0.00,0.02) | 0.13(0.05,0.24) |  | -36.75(-45.20,-27.13) | -0.30(-0.34,-0.26) |
| Saint Lucia | 0.07(0.03,0.13) | 0.13(0.05,0.25) |  | 0.03(0.01,0.07) | 0.12(0.05,0.23) |  | -48.01(-53.72,-40.26) | -0.33(-0.37,-0.28) |
| Saint Vincent and the Grenadines | 0.06(0.02,0.11) | 0.14(0.06,0.26) |  | 0.03(0.01,0.06) | 0.13(0.05,0.25) |  | -43.21(-50.54,-36.04) | -0.19(-0.23,-0.16) |
| Samoa | 0.10(0.04,0.20) | 0.15(0.06,0.28) |  | 0.11(0.05,0.22) | 0.14(0.06,0.27) |  | 9.74(-4.65,26.43) | -0.07(-0.09,-0.05) |
| San Marino | 0.01(0.00,0.01) | 0.18(0.07,0.34) |  | 0.01(0.00,0.01) | 0.18(0.08,0.33) |  | 5.10(-7.08,17.71) | -0.09(-0.11,-0.08) |
| Sao Tome and Principe | 0.10(0.04,0.18) | 0.17(0.07,0.32) |  | 0.12(0.05,0.23) | 0.16(0.06,0.30) |  | 27.53(10.82,44.33) | -0.20(-0.25,-0.15) |
| Saudi Arabia | 6.19(2.52,12.01) | 0.09(0.04,0.18) |  | 6.07(2.43,11.46) | 0.08(0.03,0.15) |  | -1.92(-13.62,12.97) | -0.58(-0.63,-0.54) |
| Senegal | 7.00(2.92,13.69) | 0.19(0.08,0.37) |  | 11.07(4.57,20.91) | 0.17(0.07,0.33) |  | 58.07(39.05,78.59) | -0.30(-0.32,-0.28) |
| Serbia | 3.32(1.34,6.33) | 0.15(0.06,0.29) |  | 1.86(0.75,3.49) | 0.14(0.06,0.26) |  | -44.12(-50.38,-35.67) | -0.38(-0.41,-0.35) |
| Seychelles | 0.04(0.01,0.07) | 0.15(0.06,0.28) |  | 0.03(0.01,0.06) | 0.13(0.05,0.25) |  | -10.95(-23.21,3.61) | -0.31(-0.36,-0.26) |
| Sierra Leone | 3.83(1.48,7.28) | 0.21(0.08,0.40) |  | 7.15(2.80,13.30) | 0.20(0.08,0.37) |  | 86.38(48.81,128.70) | -0.14(-0.16,-0.11) |
| Singapore | 1.19(0.48,2.29) | 0.18(0.07,0.35) |  | 1.47(0.62,2.70) | 0.18(0.08,0.33) |  | 23.49(7.45,41.10) | -0.07(-0.11,-0.03) |
| Slovakia | 1.84(0.73,3.47) | 0.14(0.05,0.26) |  | 1.21(0.48,2.32) | 0.14(0.06,0.27) |  | -34.18(-42.36,-24.18) | 0.09(0.05,0.12) |
| Slovenia | 0.52(0.22,0.98) | 0.13(0.05,0.24) |  | 0.40(0.16,0.76) | 0.13(0.05,0.24) |  | -24.41(-33.12,-14.11) | -0.01(-0.04,0.02) |
| Solomon Islands | 0.30(0.12,0.59) | 0.20(0.08,0.38) |  | 0.49(0.19,0.93) | 0.19(0.07,0.36) |  | 60.15(37.61,79.49) | -0.15(-0.17,-0.13) |
| Somalia | 8.39(3.10,16.58) | 0.22(0.08,0.43) |  | 22.83(9.25,45.22) | 0.22(0.09,0.44) |  | 172.03(97.90,272.56) | 0.09(0.03,0.15) |
| South Africa | 30.22(12.33,56.43) | 0.22(0.09,0.41) |  | 31.86(13.11,60.37) | 0.21(0.09,0.40) |  | 5.43(-3.93,15.79) | -0.20(-0.22,-0.17) |
| South Sudan | 5.29(2.05,10.19) | 0.20(0.08,0.39) |  | 8.31(3.32,16.02) | 0.19(0.08,0.37) |  | 57.00(30.94,94.17) | -0.15(-0.17,-0.13) |
| Spain | 9.13(3.66,16.58) | 0.12(0.05,0.21) |  | 7.39(2.99,13.83) | 0.11(0.05,0.21) |  | -19.04(-31.26,-7.22) | 0.06(-0.04,0.15) |
| Sri Lanka | 7.96(3.19,15.50) | 0.14(0.06,0.28) |  | 6.43(2.58,12.04) | 0.13(0.05,0.24) |  | -19.19(-29.34,-7.27) | -0.49(-0.51,-0.47) |
| Sudan | 9.65(3.74,19.08) | 0.11(0.04,0.21) |  | 16.36(6.84,31.10) | 0.10(0.04,0.19) |  | 69.52(50.27,95.26) | -0.29(-0.31,-0.27) |
| Suriname | 0.19(0.07,0.37) | 0.15(0.06,0.28) |  | 0.19(0.08,0.36) | 0.13(0.05,0.25) |  | 0.24(-12.96,14.36) | -0.28(-0.32,-0.24) |
| Sweden | 2.04(0.84,3.72) | 0.13(0.05,0.24) |  | 2.43(1.00,4.40) | 0.13(0.05,0.24) |  | 18.92(6.32,33.22) | -0.02(-0.09,0.04) |
| Switzerland | 2.40(0.98,4.49) | 0.21(0.08,0.39) |  | 2.74(1.14,5.07) | 0.21(0.09,0.38) |  | 14.15(1.25,29.53) | -0.05(-0.11,0.01) |
| Syrian Arab Republic | 5.42(2.18,10.46) | 0.09(0.04,0.18) |  | 2.92(1.16,5.55) | 0.08(0.03,0.15) |  | -46.06(-52.43,-36.94) | -0.47(-0.48,-0.45) |
| Taiwan (Province of China) | 5.63(2.25,10.84) | 0.10(0.04,0.20) |  | 2.67(1.08,4.98) | 0.09(0.04,0.17) |  | -52.64(-57.91,-45.37) | -0.32(-0.36,-0.27) |
| Tajikistan | 4.89(2.01,9.51) | 0.21(0.09,0.41) |  | 7.74(3.09,15.01) | 0.22(0.09,0.42) |  | 58.35(34.50,87.09) | 0.12(0.08,0.16) |
| Thailand | 24.87(9.77,48.24) | 0.15(0.06,0.29) |  | 12.56(4.92,23.85) | 0.13(0.05,0.24) |  | -49.50(-56.45,-42.45) | -0.51(-0.55,-0.46) |
| Timor-Leste | 0.64(0.26,1.23) | 0.19(0.08,0.37) |  | 0.91(0.36,1.76) | 0.17(0.07,0.34) |  | 40.46(23.43,61.25) | -0.49(-0.55,-0.43) |
| Togo | 3.58(1.40,6.94) | 0.20(0.08,0.39) |  | 6.27(2.61,12.42) | 0.19(0.08,0.38) |  | 75.04(49.09,103.77) | -0.21(-0.24,-0.19) |
| Tokelau | 0.00(0.00,0.00) | 0.15(0.06,0.30) |  | 0.00(0.00,0.00) | 0.14(0.05,0.26) |  | -41.50(-49.53,-33.15) | -0.31(-0.35,-0.27) |
| Tonga | 0.06(0.02,0.11) | 0.13(0.05,0.26) |  | 0.05(0.02,0.09) | 0.13(0.05,0.24) |  | -9.43(-21.48,2.93) | -0.08(-0.09,-0.07) |
| Trinidad and Tobago | 0.55(0.23,1.01) | 0.13(0.06,0.25) |  | 0.33(0.13,0.63) | 0.12(0.05,0.23) |  | -38.81(-46.40,-29.26) | -0.22(-0.25,-0.20) |
| Tunisia | 2.52(1.01,4.67) | 0.08(0.03,0.15) |  | 2.01(0.81,3.82) | 0.07(0.03,0.14) |  | -20.11(-29.29,-6.76) | -0.29(-0.31,-0.27) |
| Turkey | 16.71(6.85,31.89) | 0.08(0.03,0.16) |  | 13.40(5.26,25.34) | 0.07(0.03,0.14) |  | -19.80(-29.18,-7.13) | -0.39(-0.48,-0.30) |
| Turkmenistan | 3.20(1.33,6.22) | 0.21(0.09,0.41) |  | 3.23(1.28,6.11) | 0.21(0.08,0.40) |  | 1.09(-14.86,18.31) | 0.01(-0.03,0.05) |
| Tuvalu | 0.01(0.00,0.01) | 0.17(0.07,0.33) |  | 0.01(0.00,0.01) | 0.15(0.06,0.30) |  | -2.83(-17.19,14.30) | -0.29(-0.33,-0.26) |
| Uganda | 18.83(7.20,37.49) | 0.22(0.09,0.45) |  | 41.04(15.85,81.74) | 0.21(0.08,0.41) |  | 117.94(66.98,184.82) | -0.33(-0.38,-0.29) |
| Ukraine | 24.76(9.86,47.81) | 0.22(0.09,0.42) |  | 13.50(5.44,25.62) | 0.21(0.09,0.40) |  | -45.46(-52.69,-37.96) | 0.16(0.02,0.29) |
| United Arab Emirates | 0.52(0.21,0.98) | 0.09(0.03,0.17) |  | 1.04(0.42,1.94) | 0.08(0.03,0.14) |  | 97.77(74.99,123.27) | -0.27(-0.33,-0.22) |
| United Kingdom | 23.23(9.54,42.49) | 0.21(0.09,0.39) |  | 24.79(10.29,45.33) | 0.21(0.09,0.38) |  | 6.73(4.79,8.82) | -0.02(-0.03,-0.01) |
| United Republic of Tanzania | 25.31(9.44,49.46) | 0.21(0.08,0.41) |  | 48.97(19.34,94.00) | 0.20(0.08,0.39) |  | 93.50(60.53,134.08) | -0.14(-0.17,-0.12) |
| United States of America | 171.09(66.99,314.65) | 0.31(0.12,0.56) |  | 180.02(74.07,326.26) | 0.30(0.12,0.55) |  | 5.22(-12.27,27.42) | -0.04(-0.07,-0.02) |
| United States Virgin Islands | 0.04(0.01,0.07) | 0.12(0.05,0.23) |  | 0.01(0.01,0.03) | 0.11(0.04,0.20) |  | -61.69(-67.17,-56.18) | -0.28(-0.29,-0.28) |
| Uruguay | 1.78(0.75,3.30) | 0.22(0.09,0.40) |  | 1.42(0.59,2.65) | 0.22(0.09,0.40) |  | -20.23(-29.39,-9.45) | -0.07(-0.09,-0.05) |
| Uzbekistan | 18.12(7.42,33.91) | 0.21(0.09,0.40) |  | 21.64(8.57,40.86) | 0.21(0.08,0.40) |  | 19.39(0.64,41.06) | 0.05(-0.01,0.10) |
| Vanuatu | 0.11(0.04,0.21) | 0.16(0.07,0.32) |  | 0.19(0.08,0.35) | 0.16(0.07,0.30) |  | 68.99(47.37,92.64) | -0.03(-0.06,-0.01) |
| Venezuela (Bolivarian Republic of) | 9.00(3.52,17.06) | 0.13(0.05,0.24) |  | 7.73(3.04,14.83) | 0.12(0.05,0.22) |  | -14.08(-26.59,-0.93) | -0.23(-0.28,-0.19) |
| Viet Nam | 38.22(15.89,70.66) | 0.14(0.06,0.27) |  | 30.90(12.29,57.47) | 0.12(0.05,0.23) |  | -19.15(-29.44,-8.51) | -0.44(-0.48,-0.40) |
| Yemen | 7.81(3.17,14.88) | 0.11(0.04,0.21) |  | 14.17(5.68,27.47) | 0.10(0.04,0.20) |  | 81.35(57.81,106.07) | -0.16(-0.22,-0.11) |
| Zambia | 9.11(3.53,18.09) | 0.24(0.09,0.48) |  | 18.37(7.49,34.79) | 0.22(0.09,0.42) |  | 101.60(55.56,159.67) | -0.29(-0.34,-0.25) |
| Zimbabwe | 11.01(4.33,21.17) | 0.23(0.09,0.44) |  | 14.39(5.65,29.02) | 0.23(0.09,0.46) |  | 30.68(1.81,69.50) | 0.12(0.07,0.17) |
