# Supplementary material for: Epidemiological analysis of turner syndrome in children aged 0–14 years: global, regional, and national perspectives (1990-2021)
Source: Front Endocrinol (Lausanne). 2025 Apr 30;16:1552300. doi: 10.3389/fendo.2025.1552300 (PMC12074904; doi:10.3389/fendo.2025.1552300)
Supplement: Supplementary file 4 [file Table2.docx]

Table S2 Prevalence of Turner Syndrome in Children at the National Level.

| location | 1990 | |  | 2021 | |  | 1990-2021 | |
| --- | --- | --- | --- | --- | --- | --- | --- | --- |
|  | Prevalence case | Prevalence rate |  | Prevalence case | Prevalence rate |  | Cases change | EAPC |
| Afghanistan | 410.79(304.48,552.97) | 9.54(7.07,12.84) |  | 1277.86(929.85,1717.74) | 9.00(6.55,12.10) |  | 211.08(168.32,259.62) | -0.35(-0.42,-0.28) |
| Albania | 116.29(89.04,155.79) | 10.41(7.97,13.94) |  | 45.69(35.24,59.63) | 10.30(7.94,13.44) |  | -60.71(-65.50,-55.53) | -0.07(-0.13,-0.02) |
| Algeria | 726.76(545.03,981.37) | 6.78(5.08,9.15) |  | 812.74(616.42,1070.58) | 6.11(4.63,8.05) |  | 11.83(-1.82,27.52) | -0.20(-0.25,-0.15) |
| American Samoa | 1.91(1.45,2.55) | 10.06(7.62,13.40) |  | 1.36(1.02,1.84) | 9.62(7.19,12.98) |  | -28.74(-37.76,-16.96) | -0.18(-0.19,-0.16) |
| Andorra | 1.25(0.95,1.65) | 13.19(10.03,17.35) |  | 1.33(1.02,1.73) | 13.12(10.05,17.04) |  | 6.48(-7.24,22.01) | -0.04(-0.07,-0.02) |
| Angola | 747.25(549.06,1024.29) | 15.85(11.65,21.73) |  | 2242.23(1651.45,3051.42) | 14.71(10.83,20.02) |  | 200.06(164.67,247.90) | -0.25(-0.29,-0.21) |
| Antigua and Barbuda | 1.54(1.18,2.07) | 8.49(6.47,11.36) |  | 1.34(1.02,1.83) | 7.94(6.01,10.83) |  | -13.17(-24.46,-2.34) | -0.27(-0.30,-0.24) |
| Argentina | 1678.39(1283.62,2250.85) | 16.56(12.66,22.21) |  | 1640.19(1262.79,2207.59) | 16.11(12.40,21.68) |  | -2.28(-13.78,11.96) | -0.05(-0.07,-0.03) |
| Armenia | 123.33(94.22,162.29) | 11.82(9.03,15.56) |  | 66.64(51.05,87.40) | 11.25(8.62,14.75) |  | -45.97(-53.48,-37.81) | -0.13(-0.19,-0.08) |
| Australia | 319.58(244.71,418.69) | 8.44(6.46,11.06) |  | 388.73(302.30,502.21) | 8.18(6.36,10.57) |  | 21.64(6.98,37.32) | -0.07(-0.11,-0.04) |
| Austria | 201.21(157.55,258.30) | 14.92(11.68,19.16) |  | 192.28(149.54,250.38) | 14.82(11.53,19.30) |  | -4.44(-15.35,9.49) | -0.05(-0.08,-0.02) |
| Azerbaijan | 336.18(256.59,451.82) | 13.85(10.57,18.62) |  | 309.15(236.13,417.28) | 13.10(10.00,17.68) |  | -8.04(-19.86,4.94) | -0.18(-0.22,-0.13) |
| Bahamas | 7.88(5.89,10.52) | 9.76(7.31,13.04) |  | 7.40(5.66,9.81) | 9.12(6.97,12.09) |  | -6.01(-17.90,7.92) | -0.23(-0.27,-0.18) |
| Bahrain | 9.77(7.43,13.04) | 5.98(4.55,7.99) |  | 16.00(12.16,21.43) | 5.39(4.10,7.22) |  | 63.75(41.68,82.95) | -0.36(-0.40,-0.33) |
| Bangladesh | 6506.83(4800.99,8994.45) | 13.30(9.82,18.39) |  | 5270.45(3956.08,7202.67) | 11.52(8.64,15.74) |  | -19.00(-30.40,-7.76) | -0.33(-0.37,-0.29) |
| Barbados | 5.42(4.09,7.20) | 8.70(6.55,11.55) |  | 3.81(2.91,5.05) | 8.09(6.17,10.73) |  | -29.75(-38.29,-20.13) | -0.24(-0.26,-0.22) |
| Belarus | 353.57(272.73,467.36) | 14.71(11.35,19.44) |  | 221.15(167.58,287.60) | 14.01(10.62,18.22) |  | -37.45(-45.45,-28.40) | -0.04(-0.09,0.00) |
| Belgium | 371.05(291.32,478.16) | 20.54(16.13,26.47) |  | 378.63(291.22,497.50) | 19.80(15.23,26.02) |  | 2.04(-10.96,16.37) | -0.23(-0.27,-0.19) |
| Belize | 7.99(6.06,10.61) | 9.76(7.40,12.96) |  | 11.38(8.69,14.96) | 9.24(7.06,12.15) |  | 42.41(26.16,62.22) | -0.18(-0.20,-0.17) |
| Benin | 335.91(245.15,453.66) | 13.87(10.12,18.73) |  | 816.49(618.71,1095.94) | 13.43(10.18,18.02) |  | 143.07(108.80,181.79) | -0.08(-0.10,-0.06) |
| Bermuda | 0.87(0.66,1.17) | 7.29(5.58,9.81) |  | 0.53(0.41,0.70) | 6.33(4.87,8.33) |  | -38.55(-45.94,-30.08) | -0.44(-0.47,-0.42) |
| Bhutan | 32.35(24.40,43.55) | 12.34(9.31,16.61) |  | 21.25(16.22,28.45) | 11.35(8.66,15.20) |  | -34.31(-42.42,-25.46) | -0.23(-0.26,-0.20) |
| Bolivia (Plurinational State of) | 308.19(234.10,410.23) | 11.47(8.72,15.27) |  | 358.28(272.06,484.00) | 10.28(7.80,13.88) |  | 16.25(2.51,32.86) | -0.33(-0.36,-0.30) |
| Bosnia and Herzegovina | 110.72(85.70,147.71) | 10.11(7.82,13.48) |  | 50.36(38.03,66.77) | 10.27(7.75,13.61) |  | -54.52(-60.34,-48.50) | 0.02(-0.03,0.08) |
| Botswana | 95.73(70.37,130.89) | 16.21(11.92,22.17) |  | 105.83(78.47,143.48) | 15.16(11.24,20.55) |  | 10.55(-2.14,27.30) | -0.23(-0.26,-0.20) |
| Brazil | 6335.28(4863.29,8474.50) | 12.20(9.36,16.31) |  | 5872.09(4532.57,7851.07) | 12.19(9.41,16.29) |  | -7.31(-10.35,-3.89) | 0.02(0.01,0.03) |
| Brunei Darussalam | 15.17(11.70,19.53) | 16.75(12.92,21.56) |  | 15.45(11.77,20.67) | 16.34(12.44,21.85) |  | 1.85(-11.15,15.94) | -0.14(-0.17,-0.10) |
| Bulgaria | 221.30(171.77,289.80) | 12.75(9.89,16.69) |  | 130.52(100.02,172.50) | 13.37(10.25,17.67) |  | -41.02(-47.79,-32.41) | 0.11(0.02,0.19) |
| Burkina Faso | 712.11(518.74,971.31) | 15.09(10.99,20.58) |  | 1538.82(1142.47,2068.54) | 14.84(11.01,19.94) |  | 116.09(87.23,147.30) | -0.04(-0.06,-0.02) |
| Burundi | 443.59(324.76,619.37) | 16.92(12.39,23.63) |  | 934.34(684.00,1300.64) | 15.96(11.68,22.22) |  | 110.63(80.81,144.05) | -0.14(-0.16,-0.11) |
| Cabo Verde | 16.62(12.50,22.56) | 10.56(7.94,14.34) |  | 13.74(10.48,18.70) | 9.59(7.32,13.06) |  | -17.34(-27.46,-5.97) | -0.30(-0.33,-0.28) |
| Cambodia | 690.85(512.10,936.20) | 14.82(10.99,20.09) |  | 658.19(496.42,869.90) | 12.86(9.70,17.00) |  | -4.73(-17.25,8.17) | -0.46(-0.53,-0.40) |
| Cameroon | 742.41(553.65,1000.39) | 15.21(11.34,20.49) |  | 1937.49(1421.95,2598.16) | 14.39(10.56,19.29) |  | 160.97(125.48,199.08) | -0.14(-0.18,-0.10) |
| Canada | 906.54(704.62,1191.47) | 15.76(12.25,20.72) |  | 936.24(720.05,1210.93) | 15.17(11.67,19.62) |  | 3.28(-11.56,22.10) | -0.11(-0.13,-0.09) |
| Central African Republic | 227.74(166.37,313.06) | 18.63(13.61,25.60) |  | 408.76(298.78,557.79) | 17.90(13.08,24.42) |  | 79.48(56.41,109.37) | -0.16(-0.18,-0.15) |
| Chad | 464.78(343.57,637.12) | 15.88(11.74,21.77) |  | 1375.37(1015.53,1918.39) | 15.26(11.27,21.28) |  | 195.92(155.97,242.14) | -0.12(-0.15,-0.09) |
| Chile | 606.54(464.38,810.63) | 15.27(11.69,20.41) |  | 525.29(405.50,690.69) | 14.38(11.10,18.91) |  | -13.39(-24.33,0.28) | -0.18(-0.20,-0.15) |
| China | 24145.48(18329.40,32531.50) | 7.58(5.76,10.22) |  | 18642.95(14361.49,24747.09) | 7.18(5.53,9.53) |  | -22.79(-25.82,-19.34) | -0.16(-0.22,-0.09) |
| Colombia | 1050.86(802.02,1397.82) | 9.01(6.88,11.99) |  | 839.24(643.41,1112.83) | 7.91(6.06,10.49) |  | -20.14(-30.15,-8.87) | -0.41(-0.45,-0.38) |
| Comoros | 29.17(22.00,39.21) | 13.72(10.34,18.44) |  | 30.70(22.75,42.39) | 12.78(9.48,17.65) |  | 5.24(-7.31,20.91) | -0.30(-0.33,-0.27) |
| Congo | 170.64(125.98,233.44) | 16.21(11.96,22.17) |  | 285.05(208.84,385.08) | 14.77(10.82,19.96) |  | 67.04(43.23,91.87) | -0.37(-0.41,-0.32) |
| Cook Islands | 0.57(0.43,0.76) | 8.66(6.58,11.48) |  | 0.31(0.23,0.40) | 8.07(6.15,10.57) |  | -46.49(-54.31,-37.82) | -0.22(-0.26,-0.18) |
| Costa Rica | 86.79(65.86,116.12) | 7.72(5.86,10.33) |  | 73.78(56.26,96.76) | 7.25(5.53,9.51) |  | -14.99(-25.14,-2.01) | -0.15(-0.17,-0.13) |
| Croatia | 880.43(648.47,1198.23) | 15.44(11.37,21.01) |  | 1637.61(1197.46,2232.29) | 14.15(10.35,19.29) |  | -38.73(-47.07,-30.87) | -0.22(-0.24,-0.19) |
| Cuba | 92.74(72.00,122.17) | 9.40(7.29,12.38) |  | 56.82(43.76,72.99) | 9.52(7.33,12.22) |  | -33.74(-42.49,-24.54) | 0.04(0.02,0.06) |
| Cyprus | 201.62(154.72,270.53) | 8.05(6.18,10.80) |  | 133.60(102.87,179.43) | 7.52(5.79,10.10) |  | 4.75(-7.63,17.80) | -0.18(-0.22,-0.14) |
| Czechia | 28.51(21.93,37.39) | 14.40(11.08,18.89) |  | 29.87(22.87,38.69) | 13.66(10.46,17.69) |  | -21.65(-30.18,-11.02) | -0.18(-0.20,-0.17) |
| C么te d'Ivoire | 241.47(185.42,314.67) | 10.96(8.41,14.28) |  | 189.19(145.34,250.26) | 11.02(8.47,14.58) |  | 86.00(60.40,114.00) | 0.06(-0.01,0.13) |
| Democratic People's Republic of Korea | 541.96(408.37,716.73) | 9.11(6.86,12.05) |  | 414.79(312.24,556.06) | 8.69(6.54,11.65) |  | -23.46(-33.13,-11.57) | -0.13(-0.15,-0.11) |
| Democratic Republic of the Congo | 2734.48(2049.98,3753.71) | 15.45(11.58,21.20) |  | 5450.76(4053.01,7420.12) | 14.34(10.67,19.53) |  | 99.33(73.35,131.19) | -0.21(-0.25,-0.18) |
| Denmark | 113.92(86.70,148.62) | 12.90(9.81,16.82) |  | 121.52(93.81,158.88) | 12.74(9.83,16.65) |  | 6.67(-8.12,23.44) | 0.03(0.00,0.06) |
| Djibouti | 23.31(17.21,31.84) | 13.39(9.88,18.29) |  | 51.61(38.75,69.99) | 12.49(9.38,16.94) |  | 121.43(97.88,153.76) | -0.26(-0.28,-0.24) |
| Dominica | 2.34(1.77,3.12) | 9.41(7.11,12.57) |  | 1.24(0.92,1.64) | 9.04(6.75,12.00) |  | -47.05(-53.92,-39.32) | -0.16(-0.18,-0.14) |
| Dominican Republic | 274.21(210.19,373.96) | 10.17(7.80,13.87) |  | 276.75(209.61,373.57) | 9.42(7.13,12.71) |  | 0.93(-12.90,15.54) | -0.25(-0.31,-0.18) |
| Ecuador | 377.72(289.22,497.69) | 9.77(7.48,12.88) |  | 447.25(336.07,602.43) | 8.82(6.63,11.88) |  | 18.41(1.98,37.73) | -0.31(-0.33,-0.29) |
| Egypt | 1604.90(1190.55,2175.49) | 7.24(5.37,9.81) |  | 2440.28(1839.58,3309.80) | 6.62(4.99,8.98) |  | 52.05(35.29,76.55) | -0.15(-0.20,-0.11) |
| El Salvador | 210.18(160.19,280.08) | 9.74(7.42,12.98) |  | 155.84(117.71,207.28) | 8.57(6.47,11.40) |  | -25.86(-35.99,-14.53) | -0.44(-0.50,-0.38) |
| Equatorial Guinea | 31.76(23.16,43.34) | 16.13(11.76,22.01) |  | 73.24(54.02,98.69) | 12.52(9.23,16.87) |  | 130.60(97.71,165.73) | -0.96(-1.04,-0.89) |
| Eritrea | 240.31(173.48,331.64) | 15.10(10.90,20.83) |  | 364.95(272.47,491.01) | 14.46(10.79,19.45) |  | 51.86(30.23,73.91) | -0.13(-0.14,-0.12) |
| Estonia | 48.90(37.85,62.79) | 14.01(10.84,17.99) |  | 28.57(21.88,36.94) | 13.22(10.12,17.09) |  | -41.57(-49.57,-34.20) | -0.15(-0.20,-0.11) |
| Eswatini | 66.30(48.43,90.42) | 17.19(12.55,23.44) |  | 65.52(48.22,89.35) | 15.88(11.69,21.65) |  | -1.16(-15.26,13.24) | -0.25(-0.30,-0.19) |
| Ethiopia | 4017.22(2993.33,5416.92) | 16.49(12.29,22.23) |  | 6705.53(5024.57,8872.98) | 15.12(11.33,20.01) |  | 66.92(54.85,79.34) | -0.38(-0.41,-0.35) |
| Fiji | 31.29(23.66,42.90) | 11.12(8.41,15.24) |  | 29.80(22.17,40.34) | 10.93(8.14,14.80) |  | -4.76(-17.17,7.25) | -0.00(-0.03,0.02) |
| Finland | 159.02(122.21,211.59) | 16.48(12.67,21.93) |  | 133.81(103.55,174.23) | 15.80(12.22,20.57) |  | -15.85(-25.54,-4.32) | -0.11(-0.12,-0.09) |
| France | 1696.32(1431.39,2001.98) | 14.48(12.22,17.09) |  | 1760.18(1342.12,2306.49) | 15.16(11.56,19.87) |  | 3.76(-13.82,25.60) | 0.20(-0.05,0.45) |
| Gabon | 58.23(43.88,79.72) | 14.29(10.77,19.56) |  | 85.12(64.30,114.38) | 13.32(10.06,17.90) |  | 46.18(27.18,69.20) | -0.26(-0.30,-0.22) |
| Gambia | 64.99(48.41,89.67) | 14.09(10.49,19.44) |  | 131.09(99.35,179.62) | 13.20(10.00,18.08) |  | 101.73(74.42,131.18) | -0.19(-0.22,-0.16) |
| Georgia | 165.95(126.90,218.83) | 12.12(9.27,15.99) |  | 90.93(70.54,118.14) | 12.36(9.59,16.05) |  | -45.21(-51.65,-37.01) | 0.14(0.08,0.20) |
| Germany | 2415.01(1887.39,3194.83) | 18.65(14.58,24.68) |  | 2190.57(1695.94,2872.99) | 18.31(14.17,24.01) |  | -9.29(-20.82,2.79) | -0.13(-0.17,-0.09) |
| Ghana | 936.83(698.92,1271.08) | 13.95(10.41,18.92) |  | 1703.11(1259.59,2260.06) | 13.22(9.78,17.54) |  | 81.80(60.65,107.61) | -0.14(-0.16,-0.11) |
| Greece | 275.48(211.84,363.08) | 13.61(10.47,17.94) |  | 193.58(149.31,254.50) | 13.88(10.70,18.25) |  | -29.73(-38.16,-20.46) | 0.08(0.07,0.09) |
| Greenland | 3.41(2.59,4.52) | 23.96(18.22,31.76) |  | 2.62(1.96,3.43) | 22.30(16.67,29.20) |  | -23.12(-32.66,-11.65) | -0.25(-0.28,-0.23) |
| Grenada | 3.29(2.49,4.41) | 9.84(7.47,13.20) |  | 1.97(1.48,2.68) | 9.01(6.79,12.28) |  | -40.16(-47.60,-31.75) | -0.32(-0.35,-0.29) |
| Guam | 3.87(2.91,5.22) | 9.28(6.98,12.52) |  | 3.28(2.47,4.42) | 8.97(6.77,12.07) |  | -15.26(-27.27,-3.13) | -0.15(-0.22,-0.08) |
| Guatemala | 478.99(356.71,655.01) | 11.79(8.78,16.13) |  | 510.99(386.47,682.24) | 10.36(7.83,13.83) |  | 6.68(-6.72,21.01) | -0.39(-0.43,-0.34) |
| Guinea | 424.53(315.42,576.98) | 15.43(11.46,20.97) |  | 894.79(654.13,1223.00) | 14.80(10.82,20.23) |  | 110.77(85.47,142.07) | -0.15(-0.17,-0.13) |
| Guinea-Bissau | 79.83(58.14,108.60) | 16.55(12.05,22.51) |  | 139.23(102.07,191.00) | 15.50(11.36,21.27) |  | 74.41(53.32,102.85) | -0.15(-0.18,-0.11) |
| Guyana | 35.54(26.67,48.01) | 12.09(9.07,16.33) |  | 23.97(18.09,32.81) | 11.23(8.48,15.37) |  | -32.56(-40.96,-21.63) | -0.26(-0.32,-0.21) |
| Haiti | 395.81(297.31,542.29) | 14.59(10.96,19.99) |  | 582.39(435.60,791.56) | 13.38(10.01,18.19) |  | 47.14(30.36,67.15) | -0.22(-0.25,-0.18) |
| Honduras | 245.15(183.20,335.89) | 11.10(8.29,15.20) |  | 336.89(253.87,467.51) | 10.28(7.75,14.27) |  | 37.42(19.20,53.20) | -0.25(-0.27,-0.24) |
| Hungary | 188.97(145.35,246.15) | 8.87(6.82,11.55) |  | 124.81(97.24,159.33) | 8.99(7.00,11.47) |  | -33.95(-41.94,-24.10) | 0.04(-0.05,0.13) |
| Iceland | 8.62(6.57,11.09) | 13.60(10.35,17.49) |  | 9.23(7.15,12.06) | 13.66(10.59,17.86) |  | 6.96(-5.21,22.18) | 0.03(0.02,0.04) |
| India | 45284.05(34316.36,60785.04) | 13.87(10.51,18.62) |  | 45941.86(35104.78,61429.72) | 12.54(9.58,16.77) |  | 1.45(-2.03,5.22) | -0.29(-0.31,-0.27) |
| Indonesia | 9006.72(6839.56,11878.67) | 13.30(10.10,17.54) |  | 8149.58(6262.75,10857.74) | 12.11(9.31,16.14) |  | -9.52(-12.64,-6.02) | -0.34(-0.37,-0.32) |
| Iran (Islamic Republic of) | 1675.85(1282.71,2229.89) | 6.60(5.05,8.78) |  | 1191.34(919.35,1568.39) | 5.90(4.56,7.77) |  | -28.91(-31.40,-25.94) | -0.14(-0.20,-0.08) |
| Iraq | 579.59(437.13,784.70) | 7.04(5.31,9.53) |  | 858.04(660.94,1152.25) | 6.37(4.91,8.56) |  | 48.04(31.53,68.56) | -0.35(-0.38,-0.32) |
| Ireland | 186.58(147.28,234.95) | 18.99(14.99,23.91) |  | 179.07(138.21,230.96) | 17.96(13.86,23.16) |  | -4.02(-17.47,9.16) | -0.06(-0.09,-0.03) |
| Israel | 216.77(168.25,280.52) | 14.14(10.97,18.30) |  | 372.45(286.79,487.31) | 14.17(10.91,18.54) |  | 71.82(51.44,97.11) | -0.01(-0.03,0.00) |
| Italy | 1306.48(1045.92,1672.51) | 14.16(11.33,18.12) |  | 1048.78(838.22,1357.08) | 13.80(11.03,17.86) |  | -19.72(-22.24,-16.82) | -0.10(-0.11,-0.09) |
| Jamaica | 73.85(55.99,98.62) | 8.84(6.70,11.81) |  | 48.50(36.69,64.01) | 8.31(6.28,10.96) |  | -34.32(-42.47,-25.01) | -0.20(-0.25,-0.16) |
| Japan | 2926.27(2337.67,3749.32) | 12.67(10.12,16.24) |  | 1904.88(1516.40,2428.48) | 12.33(9.82,15.72) |  | -34.90(-36.81,-32.95) | -0.07(-0.09,-0.05) |
| Jordan | 99.73(76.25,133.70) | 6.11(4.67,8.19) |  | 202.07(153.99,270.57) | 5.56(4.24,7.45) |  | 102.63(77.83,134.62) | -0.36(-0.40,-0.33) |
| Kazakhstan | 740.71(571.69,1004.44) | 14.25(11.00,19.33) |  | 761.95(570.89,1030.17) | 14.04(10.52,18.98) |  | 2.87(-10.94,18.47) | -0.05(-0.08,-0.02) |
| Kenya | 1694.87(1278.07,2255.68) | 15.17(11.44,20.19) |  | 2703.62(2051.75,3599.30) | 14.48(10.99,19.28) |  | 59.52(55.69,63.43) | -0.11(-0.15,-0.06) |
| Kiribati | 4.06(3.02,5.46) | 13.74(10.24,18.48) |  | 5.38(4.00,7.20) | 12.81(9.51,17.14) |  | 32.69(15.27,54.02) | -0.16(-0.20,-0.11) |
| Kuwait | 28.41(21.48,38.06) | 5.13(3.88,6.87) |  | 39.84(30.35,52.45) | 4.71(3.59,6.20) |  | 40.22(23.01,59.68) | -0.23(-0.25,-0.22) |
| Kyrgyzstan | 239.62(181.62,322.13) | 14.28(10.83,19.20) |  | 321.80(245.73,427.77) | 14.15(10.80,18.81) |  | 34.29(17.11,56.07) | 0.02(-0.02,0.07) |
| Lao People's Democratic Republic | 287.39(215.57,393.27) | 15.59(11.70,21.34) |  | 318.05(236.27,427.17) | 13.85(10.29,18.60) |  | 10.67(-4.74,27.86) | -0.45(-0.48,-0.41) |
| Latvia | 83.96(64.29,112.48) | 14.76(11.30,19.77) |  | 41.96(32.17,56.24) | 14.13(10.83,18.93) |  | -50.02(-57.18,-42.65) | -0.01(-0.06,0.05) |
| Lebanon | 63.53(48.34,85.08) | 6.07(4.62,8.14) |  | 69.25(52.47,91.41) | 5.42(4.11,7.15) |  | 9.01(-5.51,22.65) | -0.35(-0.37,-0.32) |
| Lesotho | 118.34(87.90,163.79) | 17.34(12.88,23.99) |  | 107.22(77.35,145.67) | 17.01(12.27,23.10) |  | -9.39(-20.10,2.89) | -0.02(-0.05,0.01) |
| Liberia | 171.68(126.79,230.79) | 15.19(11.22,20.42) |  | 297.40(223.48,404.51) | 13.61(10.22,18.51) |  | 73.23(50.58,99.20) | -0.32(-0.36,-0.29) |
| Libya | 117.96(89.32,157.00) | 6.51(4.93,8.67) |  | 86.11(65.65,116.32) | 5.77(4.40,7.80) |  | -27.01(-35.10,-18.31) | -0.46(-0.49,-0.42) |
| Lithuania | 119.65(91.06,155.22) | 14.40(10.96,18.69) |  | 57.70(44.02,76.37) | 14.15(10.79,18.73) |  | -51.78(-57.60,-44.43) | 0.04(-0.04,0.12) |
| Luxembourg | 9.87(7.63,12.84) | 14.93(11.54,19.43) |  | 14.37(11.16,18.83) | 14.20(11.02,18.60) |  | 45.69(29.01,67.11) | -0.18(-0.20,-0.15) |
| Madagascar | 823.79(619.04,1121.25) | 15.10(11.35,20.55) |  | 1699.77(1279.08,2295.21) | 14.49(10.90,19.56) |  | 106.34(79.33,140.03) | -0.15(-0.18,-0.13) |
| Malawi | 794.47(579.50,1098.74) | 17.46(12.74,24.15) |  | 1284.38(942.50,1746.17) | 15.81(11.60,21.49) |  | 61.66(39.35,84.44) | -0.31(-0.36,-0.25) |
| Malaysia | 685.44(518.84,912.29) | 10.43(7.89,13.88) |  | 702.29(536.79,921.66) | 9.22(7.05,12.11) |  | 2.46(-8.19,17.02) | -0.45(-0.50,-0.40) |
| Maldives | 12.49(9.31,16.62) | 11.89(8.87,15.82) |  | 9.54(7.28,12.65) | 9.53(7.27,12.63) |  | -23.61(-34.38,-12.30) | -0.70(-0.80,-0.61) |
| Mali | 639.81(468.50,853.74) | 15.49(11.34,20.67) |  | 1717.20(1253.53,2314.58) | 14.83(10.83,20.00) |  | 168.39(134.86,206.32) | -0.15(-0.15,-0.14) |
| Malta | 12.30(9.46,15.91) | 14.05(10.81,18.18) |  | 8.92(6.90,11.71) | 13.93(10.77,18.30) |  | -27.48(-37.19,-17.53) | -0.00(-0.02,0.01) |
| Marshall Islands | 2.72(2.02,3.67) | 12.38(9.21,16.73) |  | 2.09(1.55,2.86) | 12.00(8.85,16.37) |  | -22.93(-32.63,-10.80) | -0.09(-0.13,-0.05) |
| Mauritania | 126.07(93.32,169.71) | 13.64(10.10,18.36) |  | 228.26(170.64,310.98) | 12.32(9.21,16.78) |  | 81.06(57.69,107.35) | -0.27(-0.31,-0.24) |
| Mauritius | 33.98(26.01,44.70) | 10.30(7.88,13.54) |  | 19.80(14.85,26.54) | 9.54(7.16,12.80) |  | -41.74(-48.75,-33.12) | -0.31(-0.35,-0.28) |
| Mexico | 3252.56(2502.87,4260.19) | 9.73(7.49,12.75) |  | 2830.90(2193.23,3755.58) | 8.83(6.84,11.71) |  | -12.96(-16.00,-9.83) | -0.25(-0.29,-0.22) |
| Micronesia (Federated States of) | 5.52(4.12,7.51) | 12.02(8.96,16.36) |  | 3.54(2.59,4.66) | 11.58(8.47,15.24) |  | -35.82(-43.15,-26.97) | -0.14(-0.15,-0.13) |
| Monaco | 0.49(0.38,0.64) | 13.94(10.74,18.05) |  | 0.74(0.57,0.98) | 14.86(11.51,19.72) |  | 50.54(33.27,72.48) | 0.18(0.17,0.20) |
| Mongolia | 153.39(115.59,204.97) | 17.04(12.84,22.77) |  | 176.80(133.25,237.82) | 16.27(12.26,21.89) |  | 15.26(2.34,32.85) | -0.09(-0.15,-0.03) |
| Montenegro | 15.75(12.16,20.86) | 9.74(7.53,12.91) |  | 11.33(8.68,15.08) | 10.17(7.79,13.53) |  | -28.03(-36.50,-18.71) | 0.09(0.07,0.11) |
| Morocco | 705.27(533.88,944.25) | 7.21(5.46,9.65) |  | 632.84(476.75,854.30) | 6.46(4.87,8.72) |  | -10.27(-20.92,3.77) | -0.27(-0.30,-0.24) |
| Mozambique | 1061.87(781.29,1436.73) | 17.12(12.59,23.16) |  | 2350.54(1739.09,3152.81) | 16.48(12.19,22.10) |  | 121.36(88.83,153.77) | -0.09(-0.12,-0.07) |
| Myanmar | 2129.15(1579.78,2832.26) | 14.41(10.69,19.17) |  | 1985.24(1491.22,2682.76) | 12.71(9.55,17.18) |  | -6.76(-19.21,8.15) | -0.47(-0.50,-0.45) |
| Namibia | 89.44(66.53,120.72) | 14.89(11.07,20.10) |  | 114.58(84.77,155.35) | 13.88(10.27,18.82) |  | 28.12(12.68,46.99) | -0.23(-0.26,-0.21) |
| Nauru | 0.53(0.40,0.71) | 12.55(9.36,16.79) |  | 0.48(0.35,0.64) | 11.95(8.75,16.05) |  | -10.27(-20.59,2.12) | -0.17(-0.22,-0.12) |
| Nepal | 1107.95(846.22,1481.21) | 13.15(10.04,17.58) |  | 1092.66(832.71,1477.26) | 11.84(9.02,16.01) |  | -1.38(-14.01,13.81) | -0.31(-0.33,-0.29) |
| Netherlands | 467.36(368.91,603.81) | 17.15(13.54,22.16) |  | 455.29(347.09,599.71) | 16.98(12.94,22.36) |  | -2.58(-15.47,10.02) | -0.01(-0.08,0.07) |
| New Zealand | 184.22(143.56,239.03) | 23.02(17.94,29.87) |  | 231.66(180.42,304.32) | 23.60(18.38,31.00) |  | 25.76(13.98,37.91) | 0.06(0.04,0.08) |
| Nicaragua | 165.02(125.55,219.46) | 9.06(6.89,12.05) |  | 161.87(122.85,216.19) | 8.17(6.20,10.92) |  | -1.91(-13.63,12.87) | -0.32(-0.35,-0.30) |
| Niger | 621.06(460.05,826.78) | 15.29(11.32,20.35) |  | 1865.24(1365.98,2520.77) | 14.61(10.70,19.75) |  | 200.33(162.36,244.37) | -0.19(-0.20,-0.17) |
| Nigeria | 5961.25(4535.91,7992.18) | 15.24(11.59,20.43) |  | 14368.45(10879.28,19122.50) | 14.14(10.71,18.82) |  | 141.03(134.19,147.53) | -0.18(-0.20,-0.15) |
| Niue | 0.08(0.06,0.11) | 9.83(7.32,13.14) |  | 0.04(0.03,0.05) | 9.31(7.10,12.37) |  | -54.54(-60.19,-46.32) | -0.14(-0.22,-0.06) |
| North Macedonia | 55.36(42.29,74.00) | 10.51(8.03,14.05) |  | 34.75(26.92,44.91) | 10.61(8.22,13.71) |  | -37.22(-44.20,-28.19) | 0.02(-0.02,0.05) |
| Northern Mariana Islands | 1.14(0.86,1.50) | 9.35(7.08,12.30) |  | 0.97(0.73,1.28) | 8.59(6.47,11.36) |  | -14.93(-27.08,-1.96) | -0.23(-0.28,-0.18) |
| Norway | 86.86(68.67,111.24) | 10.88(8.60,13.93) |  | 100.44(79.95,129.66) | 10.87(8.65,14.04) |  | 15.64(11.10,20.85) | 0.11(0.06,0.17) |
| Oman | 55.08(41.71,72.76) | 6.55(4.96,8.66) |  | 72.04(55.50,95.51) | 5.89(4.54,7.81) |  | 30.78(15.37,50.77) | -0.24(-0.28,-0.19) |
| Pakistan | 7004.01(5208.88,9407.39) | 14.22(10.58,19.10) |  | 11718.70(8881.13,15451.45) | 13.72(10.39,18.08) |  | 67.31(52.67,81.30) | -0.08(-0.11,-0.05) |
| Palau | 0.47(0.35,0.64) | 10.36(7.74,14.01) |  | 0.32(0.24,0.44) | 9.81(7.32,13.36) |  | -32.26(-41.09,-21.79) | -0.21(-0.24,-0.18) |
| Palestine | 59.48(45.08,79.27) | 6.14(4.66,8.19) |  | 106.19(80.30,142.10) | 5.69(4.30,7.61) |  | 78.52(58.33,102.14) | -0.23(-0.25,-0.21) |
| Panama | 70.45(54.55,96.71) | 8.45(6.54,11.60) |  | 89.22(68.67,118.53) | 7.74(5.95,10.28) |  | 26.64(11.04,42.30) | -0.22(-0.25,-0.20) |
| Papua New Guinea | 206.00(154.11,274.75) | 12.12(9.06,16.16) |  | 472.01(348.61,628.78) | 12.05(8.90,16.05) |  | 129.13(97.84,169.23) | 0.00(-0.01,0.02) |
| Paraguay | 152.37(115.03,202.10) | 9.13(6.89,12.10) |  | 169.49(131.04,225.05) | 8.44(6.53,11.21) |  | 11.24(-3.07,25.17) | -0.20(-0.22,-0.18) |
| Peru | 805.16(613.16,1082.92) | 9.70(7.39,13.05) |  | 788.25(601.55,1061.31) | 8.26(6.31,11.13) |  | -2.10(-15.80,13.85) | -0.59(-0.61,-0.56) |
| Philippines | 3097.40(2364.24,4115.84) | 12.28(9.38,16.32) |  | 3880.03(2986.53,5136.09) | 11.41(8.78,15.11) |  | 25.27(22.65,28.45) | -0.28(-0.30,-0.26) |
| Poland | 1009.06(781.19,1348.11) | 10.54(8.16,14.08) |  | 629.15(483.58,833.88) | 10.69(8.22,14.17) |  | -37.65(-40.07,-34.97) | -0.01(-0.15,0.13) |
| Portugal | 284.80(219.57,375.32) | 13.46(10.38,17.74) |  | 172.66(132.90,223.51) | 12.67(9.76,16.41) |  | -39.38(-46.70,-32.24) | -0.21(-0.24,-0.17) |
| Puerto Rico | 75.43(56.51,100.91) | 7.58(5.67,10.13) |  | 30.67(23.44,40.24) | 6.90(5.28,9.05) |  | -59.34(-64.08,-53.52) | -0.33(-0.34,-0.31) |
| Qatar | 7.42(5.69,9.83) | 5.93(4.55,7.86) |  | 26.49(20.17,36.00) | 5.36(4.08,7.29) |  | 257.12(214.03,315.98) | -0.29(-0.32,-0.25) |
| Republic of Korea | 1628.83(1283.00,2133.27) | 14.32(11.28,18.76) |  | 780.58(609.93,1019.72) | 12.85(10.04,16.79) |  | -52.08(-58.42,-44.37) | -0.35(-0.40,-0.31) |
| Republic of Moldova | 195.85(150.38,260.50) | 15.85(12.17,21.08) |  | 79.62(59.69,105.10) | 15.24(11.43,20.12) |  | -59.35(-64.90,-53.88) | 0.01(-0.05,0.07) |
| Romania | 607.55(465.53,822.23) | 10.91(8.36,14.77) |  | 329.40(256.32,437.74) | 10.94(8.52,14.54) |  | -45.78(-52.31,-37.88) | 0.04(0.01,0.06) |
| Russian Federation | 5809.45(4506.27,7662.26) | 16.74(12.99,22.08) |  | 4109.02(3175.04,5450.42) | 15.76(12.18,20.90) |  | -29.27(-31.05,-27.70) | -0.06(-0.10,-0.01) |
| Rwanda | 541.15(392.25,734.27) | 15.95(11.56,21.64) |  | 709.75(523.64,973.64) | 14.28(10.54,19.59) |  | 31.16(15.38,53.80) | -0.40(-0.45,-0.36) |
| Saint Kitts and Nevis | 1.41(1.06,1.88) | 10.02(7.53,13.34) |  | 0.89(0.68,1.19) | 9.08(6.89,12.08) |  | -36.81(-45.20,-27.13) | -0.30(-0.34,-0.26) |
| Saint Lucia | 4.82(3.62,6.46) | 9.36(7.02,12.53) |  | 2.50(1.90,3.31) | 8.43(6.41,11.13) |  | -48.10(-53.72,-40.26) | -0.33(-0.37,-0.28) |
| Saint Vincent and the Grenadines | 4.13(3.16,5.56) | 10.05(7.70,13.53) |  | 2.34(1.77,3.16) | 9.40(7.10,12.68) |  | -43.18(-50.54,-36.04) | -0.20(-0.23,-0.16) |
| Samoa | 7.56(5.70,10.25) | 10.62(8.00,14.39) |  | 8.30(6.22,10.95) | 10.38(7.78,13.70) |  | 9.72(-4.65,26.43) | -0.07(-0.09,-0.05) |
| San Marino | 0.54(0.42,0.70) | 13.22(10.29,17.17) |  | 0.57(0.44,0.74) | 12.94(9.94,16.79) |  | 5.08(-7.08,17.71) | -0.09(-0.11,-0.08) |
| Sao Tome and Principe | 6.88(5.21,9.18) | 12.15(9.20,16.20) |  | 8.76(6.56,12.03) | 11.25(8.43,15.46) |  | 27.22(10.82,44.33) | -0.21(-0.26,-0.16) |
| Saudi Arabia | 446.45(340.54,593.14) | 6.81(5.20,9.05) |  | 437.95(333.46,585.94) | 5.79(4.41,7.75) |  | -1.90(-13.62,12.97) | -0.58(-0.63,-0.54) |
| Senegal | 505.89(370.08,686.41) | 13.86(10.14,18.80) |  | 797.82(605.13,1082.05) | 12.54(9.51,17.01) |  | 57.71(39.05,78.59) | -0.30(-0.32,-0.28) |
| Serbia | 239.89(184.02,316.67) | 11.06(8.48,14.60) |  | 134.20(103.04,178.20) | 10.11(7.76,13.42) |  | -44.06(-50.38,-35.67) | -0.38(-0.42,-0.35) |
| Seychelles | 2.55(1.94,3.36) | 10.73(8.19,14.16) |  | 2.27(1.72,3.01) | 9.69(7.33,12.86) |  | -10.94(-23.21,3.61) | -0.31(-0.36,-0.26) |
| Sierra Leone | 278.58(208.77,385.05) | 15.37(11.52,21.24) |  | 515.34(383.35,692.20) | 14.41(10.72,19.36) |  | 84.99(55.24,114.65) | -0.15(-0.17,-0.12) |
| Singapore | 85.73(65.98,112.74) | 13.20(10.16,17.36) |  | 105.98(81.60,139.24) | 13.05(10.05,17.15) |  | 23.62(7.45,41.10) | -0.07(-0.11,-0.03) |
| Slovakia | 132.46(101.37,177.39) | 9.99(7.65,13.38) |  | 87.15(66.60,115.54) | 10.17(7.78,13.49) |  | -34.21(-42.36,-24.18) | 0.09(0.06,0.12) |
| Slovenia | 37.76(29.36,49.00) | 9.13(7.10,11.85) |  | 28.61(22.18,37.76) | 9.16(7.10,12.09) |  | -24.21(-33.12,-14.11) | -0.01(-0.03,0.02) |
| Solomon Islands | 21.94(16.26,29.10) | 14.09(10.45,18.69) |  | 35.22(25.99,47.45) | 13.55(9.99,18.25) |  | 60.55(37.58,79.47) | -0.15(-0.16,-0.13) |
| Somalia | 607.10(440.37,839.99) | 15.58(11.30,21.56) |  | 1665.98(1229.36,2263.84) | 16.13(11.90,21.92) |  | 174.42(137.31,216.77) | 0.08(0.03,0.14) |
| South Africa | 2190.15(1622.67,2949.34) | 16.09(11.92,21.66) |  | 2303.39(1738.43,3080.59) | 15.15(11.43,20.26) |  | 5.17(-0.13,10.61) | -0.20(-0.22,-0.17) |
| South Sudan | 384.63(288.59,519.36) | 14.66(11.00,19.79) |  | 602.88(448.63,817.83) | 14.04(10.45,19.04) |  | 56.74(37.89,82.59) | -0.16(-0.18,-0.14) |
| Spain | 659.17(531.84,809.42) | 8.41(6.79,10.33) |  | 533.92(414.87,690.18) | 8.24(6.40,10.65) |  | -19.00(-31.26,-7.22) | 0.06(-0.04,0.15) |
| Sri Lanka | 573.82(437.28,772.27) | 10.37(7.90,13.96) |  | 463.64(360.46,612.00) | 9.08(7.06,11.99) |  | -19.20(-29.34,-7.27) | -0.49(-0.51,-0.47) |
| Sudan | 696.66(518.26,945.61) | 7.83(5.83,10.63) |  | 1181.95(898.48,1594.60) | 7.12(5.42,9.61) |  | 69.66(50.27,95.26) | -0.29(-0.31,-0.27) |
| Suriname | 13.83(10.27,18.26) | 10.61(7.88,14.02) |  | 13.89(10.40,18.94) | 9.70(7.26,13.22) |  | 0.47(-12.96,14.36) | -0.28(-0.32,-0.24) |
| Sweden | 147.59(114.97,191.66) | 9.56(7.44,12.41) |  | 175.65(136.10,228.32) | 9.65(7.48,12.54) |  | 19.01(6.32,33.22) | -0.02(-0.09,0.04) |
| Switzerland | 173.03(132.58,231.46) | 14.97(11.47,20.03) |  | 197.80(150.88,257.52) | 14.84(11.32,19.33) |  | 14.31(1.25,29.53) | -0.05(-0.10,0.01) |
| Syrian Arab Republic | 390.92(296.54,522.94) | 6.60(5.01,8.83) |  | 210.99(161.98,282.00) | 5.76(4.42,7.70) |  | -46.03(-52.43,-36.94) | -0.47(-0.49,-0.45) |
| Taiwan (Province of China) | 406.18(312.48,537.84) | 7.37(5.67,9.76) |  | 192.67(147.19,259.89) | 6.54(5.00,8.82) |  | -52.57(-57.91,-45.37) | -0.32(-0.36,-0.27) |
| Tajikistan | 353.71(272.86,461.86) | 15.23(11.75,19.89) |  | 557.57(423.14,732.45) | 15.56(11.81,20.43) |  | 57.64(36.99,81.44) | 0.12(0.08,0.16) |
| Thailand | 1793.73(1354.89,2406.35) | 10.64(8.04,14.27) |  | 904.48(685.93,1190.76) | 9.26(7.02,12.19) |  | -49.58(-56.45,-42.45) | -0.51(-0.55,-0.47) |
| Timor-Leste | 46.63(34.70,63.13) | 14.02(10.43,18.98) |  | 65.41(49.58,88.03) | 12.56(9.52,16.91) |  | 40.27(22.71,61.18) | -0.50(-0.55,-0.44) |
| Togo | 258.49(188.26,351.91) | 14.67(10.68,19.97) |  | 453.46(331.02,630.87) | 13.70(10.00,19.07) |  | 75.43(48.97,103.83) | -0.21(-0.24,-0.19) |
| Tokelau | 0.07(0.05,0.09) | 10.92(8.26,14.38) |  | 0.04(0.03,0.05) | 9.82(7.28,13.45) |  | -41.55(-49.53,-33.15) | -0.31(-0.34,-0.27) |
| Tonga | 4.00(3.06,5.48) | 9.58(7.31,13.11) |  | 3.62(2.78,4.87) | 9.29(7.12,12.47) |  | -9.49(-21.48,2.93) | -0.08(-0.09,-0.07) |
| Trinidad and Tobago | 39.39(29.68,51.78) | 9.69(7.30,12.74) |  | 24.10(18.21,32.33) | 8.85(6.69,11.87) |  | -38.81(-46.40,-29.26) | -0.22(-0.25,-0.20) |
| Tunisia | 181.88(137.58,244.80) | 5.86(4.43,7.88) |  | 145.23(109.68,191.22) | 5.25(3.97,6.91) |  | -20.15(-29.29,-6.76) | -0.29(-0.30,-0.27) |
| Turkey | 1205.41(919.89,1604.82) | 5.88(4.49,7.83) |  | 968.58(747.92,1269.16) | 5.23(4.04,6.85) |  | -19.65(-29.18,-7.13) | -0.39(-0.49,-0.30) |
| Turkmenistan | 231.56(175.17,306.17) | 15.43(11.67,20.40) |  | 233.13(176.70,308.80) | 15.30(11.59,20.26) |  | 0.68(-12.25,14.77) | 0.00(-0.04,0.05) |
| Tuvalu | 0.43(0.31,0.58) | 12.29(9.00,16.73) |  | 0.42(0.31,0.55) | 11.14(8.38,14.71) |  | -2.78(-17.19,14.30) | -0.29(-0.33,-0.26) |
| Uganda | 1377.07(1004.73,1880.14) | 16.36(11.93,22.33) |  | 2972.55(2215.26,3916.05) | 14.99(11.17,19.74) |  | 115.86(89.02,148.73) | -0.35(-0.40,-0.31) |
| Ukraine | 1787.67(1372.67,2362.08) | 15.72(12.07,20.77) |  | 973.30(750.06,1309.04) | 15.34(11.82,20.63) |  | -45.56(-52.69,-37.96) | 0.16(0.02,0.30) |
| United Arab Emirates | 37.89(28.85,50.01) | 6.43(4.89,8.48) |  | 74.88(56.16,101.29) | 5.59(4.19,7.57) |  | 97.64(74.99,123.27) | -0.28(-0.33,-0.22) |
| United Kingdom | 1676.96(1332.92,2176.92) | 15.36(12.21,19.93) |  | 1790.66(1425.96,2308.47) | 15.20(12.10,19.59) |  | 6.78(4.79,8.81) | -0.02(-0.03,-0.01) |
| United Republic of Tanzania | 1836.25(1343.39,2487.62) | 15.21(11.12,20.60) |  | 3545.21(2605.05,4728.62) | 14.53(10.68,19.38) |  | 93.07(69.90,122.80) | -0.15(-0.18,-0.13) |
| United States of America | 12453.41(9831.20,16210.79) | 22.27(17.58,28.99) |  | 13109.18(10368.99,17202.00) | 22.06(17.45,28.94) |  | 5.27(1.88,8.54) | -0.05(-0.08,-0.03) |
| United States Virgin Islands | 2.69(2.04,3.59) | 8.42(6.40,11.23) |  | 1.03(0.79,1.37) | 7.69(5.89,10.23) |  | -61.72(-67.17,-56.18) | -0.28(-0.29,-0.28) |
| Uruguay | 128.73(97.95,170.01) | 15.73(11.97,20.77) |  | 102.65(78.62,136.03) | 15.56(11.92,20.63) |  | -20.26(-29.39,-9.45) | -0.07(-0.08,-0.05) |
| Uzbekistan | 1310.26(989.21,1752.40) | 15.31(11.56,20.48) |  | 1564.89(1189.67,2093.99) | 15.51(11.79,20.75) |  | 19.43(6.22,36.36) | 0.04(-0.01,0.10) |
| Vanuatu | 8.06(5.99,10.66) | 11.84(8.79,15.66) |  | 13.62(10.24,18.35) | 11.69(8.79,15.75) |  | 68.95(47.37,92.64) | -0.03(-0.05,-0.01) |
| Venezuela (Bolivarian Republic of) | 649.16(495.29,871.81) | 9.15(6.98,12.29) |  | 557.57(427.59,752.95) | 8.42(6.46,11.37) |  | -14.11(-26.59,-0.93) | -0.23(-0.27,-0.18) |
| Viet Nam | 2760.79(2121.71,3687.77) | 10.41(8.00,13.91) |  | 2230.16(1696.46,2957.60) | 9.01(6.85,11.94) |  | -19.22(-29.44,-8.51) | -0.44(-0.49,-0.40) |
| Yemen | 564.01(419.26,742.95) | 7.95(5.91,10.47) |  | 1023.08(763.71,1368.58) | 7.42(5.54,9.93) |  | 81.39(57.81,106.07) | -0.16(-0.22,-0.11) |
| Zambia | 661.27(486.16,899.47) | 17.61(12.95,23.96) |  | 1332.54(962.02,1774.97) | 16.11(11.63,21.46) |  | 101.51(76.65,128.62) | -0.30(-0.34,-0.25) |
| Zimbabwe | 794.06(590.40,1067.58) | 16.49(12.26,22.17) |  | 1045.44(771.19,1396.71) | 16.61(12.25,22.19) |  | 31.66(11.89,51.11) | 0.14(0.08,0.19) |
